# *Ucp4* Knockdown of Cerebellar Purkinje Cells Induces Bradykinesia

**DOI:** 10.1007/s12035-023-03607-1

**Published:** 2023-09-09

**Authors:** Ya-Yun Wang, Hui Liu, Shu-Jiao Li, Ban Feng, Yun-Qiang Huang, Shui-Bing Liu, Yan-Ling Yang

**Affiliations:** 1 National Teaching Demonstration Center, School of Basic Medicine, Air Force Medical University (Fourth Military Medical University), Xi’an, 710032 China; 2 State Key Laboratory of Military Stomatology, School of Stomatology, Air Force Medical University (Fourth Military Medical University), Xi’an, China; 3 Department of Human Anatomy, Histology and Embryology, Medical School of Yan’an University, Yan’an, China; 4 State Key Laboratory of Military Stomatology & National Clinical Research Center for Oral Disease & Shaanxi Engineering Research Center for Dental Material and Advanced Manufacture, Department of Pharmacy, Air Force Medical University (Fourth Military Medical University), Xi’an, China; 5 Department of Pharmacology, School of Pharmacy, Air Force Medical University (Fourth Military Medical University), Xi’an, 710032 China; 6 Department of Hepatobiliary Surgery, Xijing Hospital, Air Force Medical University (Fourth Military Medical University), Xi’an, 710032 China

**Keywords:** Mitochondrial uncoupling protein 4, Purkinje cells, Bradykinesia, Cerebellum, Movement disorders

## Abstract

Although uncoupling protein 4 (UCP4) is the most abundant protein reported in the brain, the biological function of UCP4 in cerebellum and pathological outcome of UCP4 deficiency in cerebellum remain obscure. To evaluate the role of *Ucp4* in the cerebellar Purkinje cells (PCs), we generated the conditional knockdown of *Ucp4* in PCs (*Pcp2*^*cre*^*;Ucp4*^*fl/fl*^ mice) by breeding *Ucp4*^*fl/fl*^ mice with *Pcp2*^*cre*^ mice. Series results by Western blot, immunofluorescent staining, and triple RNAscope in situ hybridization confirmed the specific ablation of *Ucp4* in PCs in *Pcp2*^*cre*^*;Ucp4*^*fl/fl*^ mice, but did not affect the expression of *Ucp2*, the analog of *Ucp4*. Combined behavioral tests showed that *Pcp2*^*cre*^*;Ucp4*^*fl/fl*^ mice displayed a characteristic bradykinesia in the spontaneous movements. The electromyogram recordings detection excluded the possibility of hypotonia in *Pcp2*^*cre*^*;Ucp4*^*fl/fl*^ mice. And the electrical patch clamp recordings showed the altered properties of PCs in *Pcp2*^*cre*^*;Ucp4*^*fl/fl*^ mice. Moreover, transmission electron microscope (TEM) results showed the increased mitochondrial circularity in PCs; ROS probe imaging showed the increased ROS generation in molecular layer; and finally, microplate reader assay showed the significant changes of mitochondrial functions, including ROS, ATP, and MMP in the isolated cerebellum tissue. The results suggested that the specific knockdown of mitochondrial protein *Ucp4* could damage PCs possibly by attacking their mitochondrial function. The present study is the first to report a close relationship between UCP4 deletion with PCs impairment, and suggests the importance of UCP4 in the substantial support of mitochondrial function homeostasis in bradykinesia. UCP4 might be a therapeutic target for the cerebellar-related movement disorder.

**Graphical Abstract:**

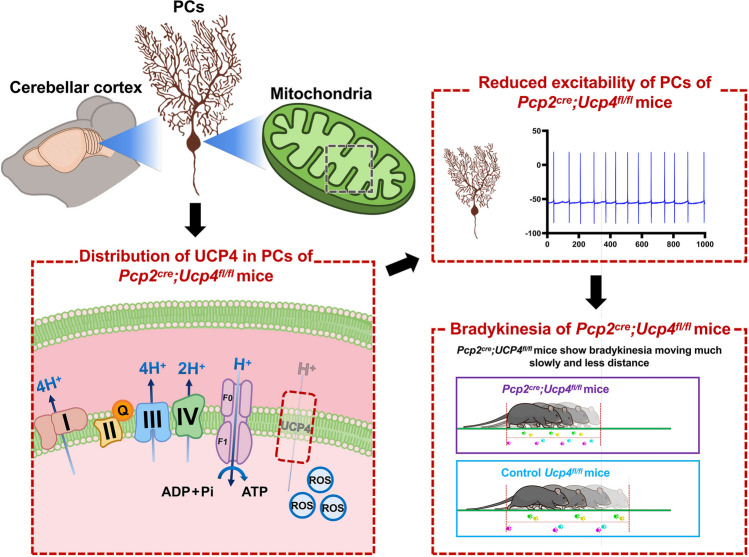

**Supplementary Information:**

The online version contains supplementary material available at 10.1007/s12035-023-03607-1.

## Introduction

Uncoupling proteins (UCPs) are the members of mitochondrial anion transporter trans-membrane protein family (SLC25s) located at the inner mitochondrial membrane (IMM) in all mammals [1–3]. UCPs uncouple electron transport from ATP synthesis by dispersing the proton gradient and play protection role against oxidative stress. Among five isoforms of UCPs (UCP1–5), UCP2, UCP4, and BMCP1/UCP5 have been reported in the brain and indicated to protect neurons from oxidative stress through diminishing the production of reactive oxygen species (ROS) [4]. Among them, UCP4 is the most abundant protein in all brain regions, including the olfactory brain (OB), cerebral cortex (CC), caudate putamen (Cpu), thalamus (Th), hypothalamus (HT), brain stem (BS), and cerebellum (CB) [5–7]. The gene *Ucp4* is located on 6p11.2 - q12 chromosomal and translates UCP4 protein with 323 amino acid and 34 KD [6]. UCP4 has been indicated to reduce the mitochondrial membrane potential (MMP), production of ROS, and oxidative stress. It has been confirmed that the knockdown of UCP4 increased MMP. On the contrary, the over-expression of UCP4 could reduce the MMP, ROS, and ATP [6–8]. It has been highlighted that the UCP4 could protect neuron against several neurodegenerative conditions like hypoxia [9] and ischemia [10], and several neurodegenerative diseases like Alzheimer’s disease (AD) [11], Parkinson’s disease (PD) [12], and schizophrenia [13]. However, the biological function of UCP4 in cerebellum and pathological outcome of UCP4 deficiency in cerebellum remains obscure.

It has been convinced that the cerebellum plays a critical role in the coordination of locomotion activity [14, 15]. Movement disruptions due to cerebellar disorders are totally different from the paralysis caused by damage to the cerebral cortex [14, 15]. Cerebellar disorders have four typical symptoms: first is hypotonia, which is present as a diminished resistance to passive limb displacements; second is astasia-abasia, which means the patient cannot stand or walk; third is ataxia, which is the abnormal execution of voluntary movements, and fourth is a tremor at the end of movement. The cerebellum constitutes only 10% of the total volume of the brain but contains more than 50% of its neuronal cells [14, 15]. The cerebellar cortex is composed with a series of similar basic microcircuits [14, 15], and contains three layers: the external molecular layer (ML), the middle Purkinje cell layer (PCL), and the internal granular cell layer (GCL) [16]. There are 6 main neuronal populations including Purkinje, stellate, basket, Lugaro, Golgi, and granule cells in the cerebellar cortex. The granule cells are glutamate-expressing excitatory neurons, and others are GABA-expressing inhibitory neurons. Of these, Purkinje cells (PCs) are considered to be the most functionally important because they are the only projection neurons of the cerebellar cortex [16]. PCs have larger somatic bodies with 50–80 μm in diameter, and send the huge dendritic trees into the ML contacting climbing fibers and parallel fibers. And PCs send long axons through GCL and make inhibitory synaptic contacts with neurons of the cerebellar deep nuclei within the cerebellar white matter to control movement [16]. Multiple gene deletions in PCs resulted in PCs dysfunction and further motor disorders. Tsuda et al. have reported that the deletion of the zinc finger transcription factor Gfi1 in PCs of mice could cause PCs degeneration and mimic spinocerebellar ataxia type 1 (SCA1) [17]. Liu et al. have shown that the mice with conditional knock-out of 3-phosphoinositide-dependent protein kinase-1 (PDK1) in PCs displayed impaired motor balance and their PCs showed the reduced spontaneous firing [18]. Zhou et al. have reported that the mice with PCs ablation of transferrin receptor 1 (TFR1) induces ataxia, but does not affect social behaviors [19]. However, the roles of *Ucp4* and the result of *Ucp4* dysfunction in the PCs have not been investigated so far.

To evaluate the role of *Ucp4* in cerebellar PCs, we first generated conditional knockdown of *Ucp4* in PCs (*Pcp2*^*cre*^*;Ucp4*^*fl/fl*^ mice) by breeding *Ucp4*^*fl/fl*^ mice with *Pcp2*^*cre*^ mice. Second, the specific knockdown of *Ucp4* and the reservation of *Ucp2*, the analog of *Ucp4*, were confirmed by Western blot, and triple RNAscope in situ hybridization. Third, the combined behavioral tests of rotarod test, open field (OF) test, CatWalk analysis, and elevated plus maze (EPM) showed a characteristic bradykinesia with the reduction of spontaneous movements in *Pcp2*^*cre*^*;Ucp4*^*fl/fl*^ mice. Fourth, the *Pcp2*^*cre*^*;Ucp4*^*fl/fl*^ mice did not show hypotonia by electromyogram recordings (EMG) detection on gastrocnemius muscle. Fifth, the electrical patch clamp recordings showed the altered properties of both spontaneous and evoked firings in PCs of *Pcp2*^*cre*^*;Ucp4*^*fl/fl*^ mice. Sixth, the knockdown of *Ucp4* resulted in the mitochondrial impairments reflected by the significant increases of ROS generation and increased mitochondrial circularity in cerebellum of *Pcp2*^*cre*^*;Ucp4*^*fl/fl*^ mice. The present study indicates a close relationship between UCP4 deletion with PCs impairment, and suggests the importance of UCP4 in the substantial support of mitochondrial function homeostasis in bradykinesia. UCP4 might be a therapeutic target for the cerebellum-related movement disorder.

## Materials and Methods

### Animals and Experimental Design

*Pcp2* ^*cre*^ mice were purchased from Jackson Laboratory (Stock No: 004146, America). *Ucp4*^*fl/fl*^ mice were purchased from Cyagen (Serial number: CKOCMP-74011-Slc25a27, China). All surgical experiments were performed under pentobarbital. The mice were housed on a 12-h light-dark cycle. All the experiments in the present study were performed according to the ethical guidelines of the International Association for the Study of Pain and approved by the Air Force Medical University Committee on Animal Care and Use (IACUC-20190107). All efforts were made to minimize the number of animals used and animal suffering.

The experimental design is shown in Fig. S1. First, we generated conditional knockdown of *Ucp4 *in Purkinje cells (PCs) (*Pcp2*^*cre*^*;Ucp4*^*fl/fl*^ mice) by breeding *Ucp4*^*fl/fl*^ mice with *Pcp2*^*cre*^ mice and the genotype was confirmed by polymerase chain reaction (PCR). Second, the specific ablation of *Ucp4* in PCs was confirmed by Western blot, double immunofluorescent staining, and triple RNAscope in situ hybridization. Third, the behavioral characteristics of *Pcp2*^*cre*^*;Ucp4*^*fl/fl*^ mice was evaluated by rotarod test, open field (OF) test, CatWalk analysis, and elevated plus maze (EPM). Fourth, it was analyzed whether or not the behavioral activities could be deteriorated by harmaline-induced tremor application. Fifth, whether or not the hypotonia was present in *Pcp2*^*cre*^*;Ucp4*^*fl/fl*^ mice was studied by electromyogram recordings (EMG) detection on the gastrocnemius muscle. Sixth, the spontaneous or evoked firings properties of PCs of *Pcp2*^*cre*^*;Ucp4*^*fl/fl*^ mice were studied by the electrical patch clamp recordings. Seventh, the mitochondrial impairment in cerebellum tissues of *Pcp2*^*cre*^*;Ucp4*^*fl/fl*^ mice were evaluated by the levels of ATP, MMP and ROS.

### Generation Strategy and Genotype

The strategy employed to generate conditional ablation of *Ucp4* within PCs was to breed *Ucp4*^*fl/fl*^ mice with *Pcp2*^*cre*^ mice. *Pcp2*^*cre*^ mice were purchased from Jackson Laboratory (Stock No: 004146, America), in which a *Cre* recombinase sequence was exclusively expressed in PCs [20, 21]. *Ucp4*^*fl/fl*^ mice with the *Ucp4* exon 3 flanked by loxP sites were purchased from Cyagen (Serial number: CKOCMP-74011-Slc25a27, China). In the P0 generation, *Pcp2*^*cre*^ mice and *Ucp4*^*fl/fl*^ mice were crossed to generate *Pcp2*^*cre*^*;Ucp4*^*fl/+*^ mice as F1, in which half of the PCs expressed Cre recombinase to cut the *Ucp4 loxp* sequence, while the remainder still expressed one *Ucp4* allele with a *loxP* site. Then, F1 *Pcp2*^*cre*^*;Ucp4*^*fl/+*^ mice were crossed with *Ucp4*^*fl/fl*^ mice to generate *Pcp2*^*cre*^*;Ucp4*^*fl/fl*^ mice as F2. In *Pcp2*^*cre*^*;Ucp4*^*fl/fl*^ mice, almost all PCs lost the *Ucp4* sequence attributing to *Cre* recombinase cutting *loxP* sites, while non-PCs expressed the *Ucp4* sequence with a *loxP* site.

There were five genotypes of mice in the present study: (1) *Pcp2*^*cre*^ mice, which expressed two bands of 567 bp for *Cre* and 119 bp for the *Ucp4* allele without *loxP*; (2) *Ucp4*^*fl/fl*^ mice, which only expressed one band of 187 bp for the *Ucp4* allele with loxP; (3) *Pcp2*^*cre*^*;Ucp4*^*fl/+*^ mice, which expressed three bands of 567 bp for the *Cre* site, 187 bp for the *loxP* site, and 119 bp for the *Ucp4-wide* type; (4) *Pcp2*^*cre*^*;Ucp4*^*fl/fl*^ mice, which showed two bands of 567 bp and 187 bp by PCR; and (5) *Ucp4*^*fl/+*^ mice, which expressed two bands of 187 bp for the *Ucp4* allele with the *loxP* site and of 119 bp for the *Ucp4* allele with no *loxP* site. The mouse genotype was identified by polymerase chain reaction (PCR) with genomic DNA obtained from the tails. The primers and the strategy used for genotypes identification are shown in Table 1 and Table 2, respectively. The PCR program was as follows: 94°C for 5 min, 35 cycles of 94°C for 30 s for denaturation, 62°C for 30 s for annealing, and 72°C 20 s for elongation. The primers and PCR conditions were designed by Tsingke Biotechnology Co., Ltd.

**Table 1:** The primers used for genotype identification

Primer	Sequences (5′-3′)
P1	CACCAGTCTTAGTTACACAAATG
P2	TGAATGGTAACCAAATAAGAGGC
P3	ATTCTCGTGGAACTGGATGG
P4	GGACAGGTAATGGTTGTCTGG

The PCR program used was as follows:

94°C for 3 min, then 35 cycles of 94°C for 30 s for denaturation, 62°C for 35 s for annealing, and 72°C 45 s for elongation.

**Table 2:** The strategy for genotype identification

Mice type	Combination of primers for genotyping	Number and length of product
*Pcp2*^*cre*^	P3+P4	One band with 567 bp
*Ucp4*^*fl/fl*^	P1+P2	One band with 187 bp
*Pcp2*^*cre*^*;Ucp4*^*fl/+*^	P1+P2+P3+P4	Three bands with 119 bp, 187 bp, and 567 bp
*Pcp2*^*cre*^*;Ucp4*^*fl/fl*^	P1+P2+P3+P4	Two bands with 187 bp and 567 bp

Hence in the next a serious of experiments, *Pcp2*^*cre*^*;Ucp4*^*fl/fl*^ mice were used as the experimental group, and *Ucp4*^*fl/fl*^ mice were used as control group.

### Western Blot

To confirm successful knockdown of UCP4 in *Pcp2*^*cre*^*;Ucp4*^*fl/fl*^ mice, the cerebellar expression levels of UCP4, as well as the mitochondrial fission factor dynamin-related protein 1 (DRP1) and two mitochondrial fusion factors of mitofusion 2 (MFN2) and optic atrophy 1 (OPA1), were detected by Western blot. Twenty micrograms of fractionated protein extracts from cerebellum tissues was loaded on 10% acrylamide gel and blotted onto a methanol-activated PVDF membrane (Millipore, USA). Immunoblots were soaked in 5% nonfat milk 2 h at room temperature and subsequently probed with primary antibodies overnight at 4 °C and then incubated with corresponding secondary antibodies. The antibodies used for Western blot are described in Table 3. The bands were detected with enhanced chemiluminescence (Beyotime, China) followed by exposure to luminometer (Bio-Rad, USA) and analyzed by ImageJ software. Target protein levels were normalized against GAPDH levels and expressed as fold changes relative to those of the naive control group.

**Table 3:** The antibodies used for Western blot

Antibody type	Antibody name	Company	Product number	Dilution condition	Species
Primary antibody	Anti-UCP4	Invitrogen	PA5-116568	1:1000	Rabbit
	Anti-UCP4	Santa Cruz Biotechnology	#sc-365295	1:200	Mouse
	Anti-Drp1	Cell Signaling Technology	#8570	1:1000	Rabbit
	Anti-OPA1	Santa Cruz Biotechnology	#sc-393296	1:1000	Rabbit
	Anti-Mfn2	Cell Signaling Technology	#9482	1:1000	Rabbit
	Anti-GAPDH	Cell Signaling Technology	#5174	1:1000	Rabbit
	Anti-beta Actin	Engibody	AT0001	1:5000	Mouse
Secondary antibody	HRP, goat anti-mouse IgG	Abbkine	#A21010	1:5000	Goat
	HRP, goat anti-rabbit IgG	Abbkine	#A21020	1:5000	Goat

### Immunofluorescent Staining

To confirm the specific knockdown of UCP4 within the PCs of *Pcp2*^*cre*^*;Ucp4*^*fl/fl*^ mice, the double immunofluorescent staining of UCP2, or the UCP4 analog of UCP2, with the PCs marker calbindin was performed, according to the methods described previously [18]. *Pcp2*^*cre*^*;Ucp4*^*fl/fl*^ mice were used as the experimental group (male, 8-weeks-old, *n* = 6), and *Ucp4*^*fl/fl*^ mice (male, 8-weeks-old, *n* = 6) were used as control group. The mice were anesthetized and transcardially perfused with 4% paraformaldehyde (PFA) in PBS. The brain was removed and postfixed in 4% PFA overnight at 4°C, and subsequently immersed in 30% sucrose solution for 2 days. Half of the brain was sagittally sectioned at 30-μm thicknesses on a cryostat (Leica CM1850, Germany) and used for double immunofluorescent staining; and another half of the brain was sagittally sectioned at 10-μm thicknesses on a cryostat for triple RNAscope in situ hybridization. In double immunofluorescent staining, the mounted sections were permeabilized with 0.3% TritonX-100 at room temperature for 1 h. The primary and secondary antibodies are described in Table 4. A confocal laser microscope (FV1000; Olympus, Tokyo, Japan) was used to observe digital images. Twelve slices obtained from 6 mice in each group were randomly chosen. Images were analyzed by individuals blinded towards the experimental groups. The photography view field focused on the 4/5 lobes of the cerebellar cortex (4/5Cb). Anatomical structures were analyzed according to the Fourth Edition of Paxinos and Franklin The Mouse Brain Atlas [22] and Allen map (http://mouse.brain-map.org/static/atlas). According to the previous report [18], the total number of PCs was obtained by drawing the outline of Purkinje cell layer (PCL) in the selected 4/5Cb of cerebellum and counting the number of PCs in the lobule manually. The length of the PCL was then measured, and the density of PCs was calculated by dividing the total number of PCs by the length of PCL.

**Table 4:** The antibodies used for immunofluorescent staining

Antibody type	Antibody name	Company	Product number	Dilution condition	Species
Primary antibody	Anti-UCP4	Santa Cruz Biotechnology	#sc-365295	1:50	Mouse
	Anti-UCP2	Santa Cruz Biotechnology	#sc-390189	1:50	Mouse
Secondary antibody	Dylight 488, goat anti-mouse IgG	Abbkine	A23210	1:200	Goat
Alexa Fluor® 594 Conjugate	Anti-calbindin	Cell Signaling Technology	#88831	1:50	Rabbit
Nucleus dye	DAPI	Beyotime	C1005	1: 1000	-

### Triple RNAscope In Situ Hybridization

To further confirm the specific knockdown of *Ucp4* within the PCs of *Pcp2*^*cre*^*;Ucp4*^*fl/fl*^ mice, the triple RNAscope in situ hybridization was performed by using the probes of *Ucp4*, *Ucp2*, and the PCs marker *Pcp2*, according to the methods described previously [23]. The steps before sectioning were the same as that in double immunofluorescent staining. In brief, sagittal sections were cut (10 μm) with cryostat and the tissues were adhered to SuperFrost Plus charged slides. The slices were thawed briefly to adhere to the slides, but they were immediately returned to the −20 °C cryostat chamber until the slices were completed. After the slides were baked at 37°C for 3 h, the brain was washed with 0.01 M PBS for 5 min. Each slide was incubated with hydrogen peroxide (322281, ACD, USA) at room temperature for 10 min. After washing twice in distilled water, the slides were boiled with the Target Retrieval reagents (322000, ACD, USA) at 97°C for 10 min. Immediately, the tissue was placed in distilled water at room temperature and then dehydrated with absolute ethanol for 3 min. Next, the slides were air-dried and boundaries were drawn round each tissue with a hydrophobic pen (CIRISC PAP pen, I.S. CIRCLE WRITER, Japan). When the hydrophobic boundaries were completely dried, protease III reagent (322281, ACD, USA) was added to each tissue until it was completely covered. Subsequently, the slides were incubated in a preheated HybEZ oven (ACD, USA) at 40°C for 30 min. These tissues were used for hybridization. A mixture of three probes was then added to each slide until the tissue was fully covered. The probes used in RNAscope in situ hybridization are shown in Table 5. Positive control probes for low-, medium-, and high-expressing housekeeping genes (POLR2A, PPIB, and UBC, respectively), as well as negative control probe for DapB, were used. After incubating for 2 h in a HybEZ oven at 40°C, the slides were washed twice in 1× RNAscope® washing buffer (310091, ACD, USA) for 2 min each time. The slides were returned to the oven for 30 min following submersion in AMP-1 reagent. This step was repeated with AMP-2 and AMP-3 reagents for 30 min and 15 min, respectively. The HRP-C1, HRP-C2, and HRP-C3 signals were processed. Opal 520 (ASOP520, ASbio, USA) was applied to mark the channel 1 probe, Opal 690 (ASOP690, ASbio, USA) was applied to mark the channel 2 probe, and Opal 570 (ASOP570, ASbio, USA) was applied to mark the channel 3 probe. Finally, the tissue was submerged with Prolong Gold Antifade Mountant with DAPI. High-resolution imaging was performed using confocal microscopy (FV3000, Olympus). The number (No) of *Ucp4* mRNA-dots (violet) and *Ucp2* mRNA-dots (green) on three layers of cerebellar cortex including ML, PCL, and GCL was calculated. The intensity of *Pcp2* mRNA-dots (red) on three layers was calculated. According to the previous report [24], we counted the number of UCP4 mRNA-dots manually in confocal monolayer imaging. The thickness of each slice was 30 microns. The calculated area of ML and GCL are 40 and 100 μm^2^, respectively.

**Table 5:** The probes used in RNAscope in situ hybridization

Name	Mitochondrial localization	Function	Accession number	Target region	Dilution	TSA® Plus channel
*Pcp2*	-	Purkinje cell marker	NM_001129804.1	2–302	1:50	2
*Ucp2*	IMM	Mitochondrial uncoupling	NM_011671.5	2–1002	1:1	1
*Ucp4*	IMM	Mitochondrial uncoupling	NM_028711.4	457–1410	1:50	3
*Polr2a*	Positive control	-	NM_009089.2	2802–3678	1:1	1
*PPIB*	Positive control	-	NM_011169.2	98–856	1:1	2
*UBC*	Positive control	-	NM_019639.4	34–860	1:1	3
*DapB*	Negative control	-	EF191515	414–862	1:1	1, 2, 3

*Ucp4*, uncoupling protein 4; *Ucp2*, uncoupling protein 2; *Pcp2*, Purkinje cell protein 2; *IMM*, inner mitochondrial membrane.

### Combined Behavioral Tests

Then, the combined behavioral tests of rotarod test, open field (OF) test, CatWalk analysis, and elevated plus maze (EPM) were used to evaluate the effect of the specific knockdown of UCP4 within the PCs on the spontaneous movements of mice. *Pcp2*^*cre*^*;Ucp4*^*fl/fl*^ mice were used as the experimental group (male, 8-weeks-old, *n* = 6), and *Ucp4*^*fl/fl*^ mice (male, 8-weeks-old, *n* = 5) and *Pcp2*^*cre*^*;Ucp4*^*fl/+*^ mice (male, 8-weeks-old, *n* = 6) were used as control group. The mice were recorded in order of OF test for 15 min, EPM for 5 min, CatWalk for 5 min, and Rotarod test for 5 min.

#### Open Field Test

To measure the freely spontaneous movement of the transgenic mice, the mice were placed in the center of a 50 × 50 × 50.5 cm opaque square box (DigBehav, Jiliang) and their autonomous movements were recorded for 15 min by a camera connected to a computer, according to the methods described previously [25]. The movement of the mice was tracked automatically and analyzed by any-maze software. Four parameters were analyzed: (1) the total distance (m); (2) average speed (cm/s); (3) the ratio of the length of the moved path in the 4 central squares to the length of the moved path in the 12 peripheral squares (Ratio of Path_cent_. to Path_prl_.); (4) the ratio of the time used in the 4 central squares to the time used in the 12 peripheral squares (Ratio of Time_cent__._ to Time_prl__._).

#### CatWalk Analysis

The Noldus CatWalk analysis is very useful to evaluate the gait of mice, so the mice were in the entrance of a 1.3-m-long glass walkway, according to the methods described previously [26–28]. The mice were allowed to walk freely across the runaway to reach the goal box. Paw prints and silhouettes were captured by a digital high-speed video camera. Three to five compliant walkway trials were recorded for each mouse and after each trial, the walkway was cleaned with 40% ethanol. CatWalk XT 10.1 software (Noldus, Netherlands) was used to analyze the data. The training was conducted thrice a day for 3 consecutive days, and the experiment was formally conducted on the 4th day. Seven parameters of gait analysis were analyzed: (1) the regularity index (RI %), a fractional measure of inter-paw coordination which was calculated as the number of normal step sequence patterns relative to the total number of paw placements; (2) the base of support (BOS), which was the average width (cm) between either the hind paws (BOS_*HP*_) or the front paws (BOS_*FP*_); (3) the print area (cm^2^), which was the print size of the left front paw (LF), left hind paw (LH), right front paw (RF), and right hind paw (RH), respectively.

#### Rotarod Test

To measure the balance and coordination of the transgenic mice, the mice were placed on a 3-cm diameter accelerated rotarod (BZY007, Jiliang, Shanghai, China), according to the methods described previously [29]. The rotarod started rolling at 4 rpm and increased to 40 rpm in 1 min, and maintained for 4 min. Each test was conducted for 5 min. The training was conducted thrice a day for 3 consecutive days, and the experiment was formally conducted on the 4th day. The latency to fall from the rotarod was recorded.

#### Elevated Plus Maze

To measure the emotional activities of the transgenic mice, the mice were placed in the elevated plus maze (EPM) (DigBehav) and their autonomous movements were recorded for 5 min by a camera connected to a computer, according to the methods described previously [30]. The EPM instrument consisted of a common central platform (5 × 5 cm^2^) with two open arms (30 × 5 cm^2^) and two closed arms (30 × 5 × 15 cm^3^) extending from it, which was elevated to a height of 38 cm above the ground. The two closed arms were surrounded by opaque walls. The mice were placed alone on the central platform, facing the open arms, and they were freely tracked under an overhead camera for 5 min. Six parameters were analyzed: the duration and frequency in the closed arms (Duration_closed_ and Frequency_closed_), the duration and frequency in central platform (Duration_cent._ and Frequency_cent._), and the duration and frequency in open arms (Duration_open_ and Frequency_open_).

### Electromyogram Recording

Considering the hypotonia is a typical symptom of cerebellar disorders, in the next step, we analyzed whether or not the muscle damage was present in *Pcp2*^*cre*^*;Ucp4*^*fl/fl*^ mice by using electromyogram recording (EMG) applied on the gastrocnemius muscle. *Pcp2*^*cre*^*;Ucp4*^*fl/fl*^ mice were used as the experimental group (male, 8-weeks-old, *n* = 3), and *Ucp4*^*fl/fl*^ mice (male, 8-weeks-old, *n* = 3) and *Pcp2*^*cre*^*;Ucp4*^*fl/+*^ mice (male, 8-weeks-old, *n* = 3) were used as control group. The mice were anesthetized with 3% isoflurane gas and maintained during EMG recording at 2% isoflurane gas, according to the methods described previously [31, 32]. The mice were immobilized and the hair around the gastrocnemius muscle of the right hind limb was removed. Four EMG electrodes, two stimulation electrodes, and two leading electrodes were inserted approximately 3–5 mm apart into the tricep muscle of the gastrocnemius of the right hind limb to record the bioelectric potential difference across the muscle tissue. The yellow electrode (positive electrode, +) and the green electrode (negative electrode, –) were made up of stimulation electrodes, while the leading electrodes were composed of a red electrode (positive electrode, +) and a black electrode (negative electrode, –). The positive stimulation electrode was inserted into the medial side of the beginning of the gastrocnemius muscle, and the negative stimulation electrode was inserted into the lateral side. Then, we inserted the negative leading electrode 4 mm below the positive stimulation electrode. In the next step, the positive leading electrode was inserted 4 mm below the negative leading electrode. Finally, we inserted a ground electrode (gray electrode) below the negative stimulation electrode. When recording the EMG, we first recorded the signal baseline for 20 s, before performing stimulation for 15 s at 1 V and 50 Hz to record a new signal baseline lasting 20 s. EMG data were analyzed using the BL-420N Biological function test system (Techman, Chengdu, China). Raw EMG data were filtered using a 2 KHz bandpass filter.

### Electrical Patch Clamp Recordings

To study whether or not the conditional knock-out of Ucp4 in PCs could impair the spontaneous or evoked firings properties of PCs, we performed the electrical patch clamp recordings, according to the methods described previously [33]. *Pcp2*^*cre*^*;Ucp4*^*fl/fl*^ mice were used as the experimental group (male, 8-weeks-old, *n* = 4), and *Ucp4*^*fl/fl*^ mice (male, 8-weeks-old, *n* = 4) and *Pcp2*^*cre*^*;Ucp4*^*fl/+*^ mice (male, 8-weeks-old, *n* = 4) were used as control group. The mice were anesthetized by intraperitoneal administration with pentobarbital and immediately sacrificed. The mice cerebella were cut into 300-μm-thick sagittal slices on a vibrating microtome (Leica VT 1200s) at 4°C. Then, the slices were transferred into ice-cold and oxygen (95% O_2_ and 5% CO_2_) artificial cerebrospinal fluid (ACSF) at room temperature for recording. The ACSF contained 124 mM NaCl, 4.4 mM KCl, 2 mM CaCl_2_, 1 mM MgSO_4_, 25 mM NaHCO_3_, 1 mM NaH_2_PO_4_, 10 mM glucose, with a final osmolality of 320 mOsm and pH 7.4.

First, the spontaneous action potential (AP) firing of PCs was recorded in the current clamp mode, without additional current injections. Seven parameters were measured: (1) the threshold potential (mV) defined as the first point to which the membrane potential depolarized when an action potential occurs and reflects the excitability of cells; (2) action potential (AP) peak (mV) defined as the maximum voltage of the AP; (3) the half-width of the AP (ms) defined as the duration measured at one half of the AP peak above the threshold potential; (4) the rise slope (mV/ms) and (5) decay slope (mV/ms) defined as the value by which the voltage increases or decreases per unit time; (6) the frequency (Hz) defined as the number of spikes per unit time; (7) afterhyperpolarization (AHP, mV) defined as the peak voltage after the AP.

Second, the evoke AP was made with depolarizing currents of 0–100 pA (500 ms duration, step 10 pA). Rheobasing was defined as the minimum current required to evoke spontaneous APs. Three types of PCs electrical properties were classified: (1) active type meaning the PCs which showed spontaneous firing and they displayed the increasingly evoked spike numbers by the increasing strengths of stimulation; (2) inactive type meaning the PCs which did not show spontaneous firing began to display the evoked spikes, and their evoked spike numbers increased with the increased strengths of stimulation; (3) quiescent type meaning the PCs which did not show spontaneous firing and failed to display the evoked spikes whatever the increased strengths of stimulation. And the number of 3 types of PCs was calculated.

After recording, biocytin (0.5%) was introduced into the recording region to label the morphology of the recorded neurons. Then, the brain slices were immediately fixed in 4% PFA in PBS for 4 h at room temperature. Sections were then transferred to 0.01 M PBS containing 1% Triton X-100 (PBS-TX) and stored at 4°C. The sections were then blocked with 3% normal bovine serum for 30 min. After thoroughly washing with PBS, the tissue sections were incubated with a PBS solution containing a mixture of Alexa Fluor®594 streptavidin and calbindin (D1I4Q) XP® Rabbit mAb (Table 4) for 18 h.

### Transmission Electron Microscope Analysis

According to our previous publication [34], 4 mitochondrial parameters of density, size, perimeter, and circularity were calculated and compared between *Ucp4*^*fl/fl*^ mice and *Pcp2*^*cre*^*;Ucp4*^*fl/fl*^ mice. In brief, mice were anesthetized and then perfused with precooled PBS. Cerebellum was removed on ice immediately. After that, 2.5% glutaraldehyde was sequentially fixed at 0.1 M PBS (pH 7.4) at 4°C for 4 h, washed with 0.1 M PBS for 3 times, fixed at 1% oric acid at 4°C for 2 h, washed with 0.1 M PBS for 3 times. The samples were then dehydrated in 50% ethanol, 70% ethanol, and 90% ethanol for 15 min. Finally, it is dehydrated in 100% acetone (3 × 15 min) and embedded in resin. The 50 nm slices were then collected on a copper mesh and stained with lead nitrate and uranyl acetate for 10 min. Images were taken using a transmission electron microscope (TEM; JEM 1400, Olympus, Japan). The mitochondrial morphology was analyzed by ImageJ. By using the freehand selections of this software, the outline of mitochondria was carefully drawn, and then the perimeter and area of the mitochondria were measured. The circularity of mitochondrial was determined according to the method that Zhang et al. described, that is, circularity = 4 π area / perimeter^2^.

### Reactive Oxygen Species Probe Imaging

According to the standard protocol of BestBio’s instructions, mice were sacrificed by neck dissection. The brain tissue was removed, cut into 15 m thick at −20°C and mounted on glass slides. ROS probe (DHE) covered the tissue (1:500) at 37°C for 30 min. After sealing, the slides were promptly detected under confocal fluorescence microscope (FV3000, Olympus, Japan) for fluorescence detection. The images were observed at 610 nm wavelength.

### Measurements of ROS Generation, Mitochondrial Membrane Potential, and ATP Level in Mitochondrial Purification Isolated from Cerebellum by Microplate Reader

In general, *Pcp2*^*cre*^*;Ucp4*^*fl/fl*^ mice were used as the experimental group (male, 8-weeks-old, *n* = 3), and *Ucp4*^*fl/fl*^ mice (male, 8-weeks-old, *n* = 3) and *Pcp2*^*cre*^*;Ucp4*^*fl/+*^ mice (male, 8-weeks-old, *n* = 3) were used as control group. First, the mitochondria isolation from extracted cerebellum tissues was performed using the Mitochondria Purification Kit (SM0020, Solarbio, Beijing, China). Second, the following three assays were performed on a Microplate reader (Spark, Tecan, Switzerland).

#### ROS Generation

ROS generation was measured in the mice’s cerebellum tissue using the 2,7-dichlorodi-hydrofluorescein diacetate (DCFH-DA) Assay Kit (S0033S, Beyotime, China). The purified mitochondria were incubated with 20 μM DCFH-DA in serum-free medium for 20 min at 37°C in the dark. After washing serum-free medium by DMEM, the ROS fluorescence was measured by 488 nm of excitation wavelength and 525 nm of emission wavelength.

#### MMP Measurement

MMP measurement was measured on the isolated mitochondria using JC-1 Assay Kit (C2006, Beyotime, China). The fluorescence was measured by 485 nm of excitation wavelength and 590 nm of emission wavelength.

#### Detection of ATP

The amount of ATP was determined using the enhanced ATP assay kit (S0027, Beyotime Biotechnology, China). The concentration of ATP depends on the standard curve and was normalized to cells which was figured out by cell counter.

### Statistical Analysis

Statistical comparison of each experiment was presented in the figure legend. The data were shown by mean ± SD. All data was firstly analyzed for the normality test and homogeneity of variance test. For three samples comparison, one-way ANOVA was used to assess differences between groups and the LSD post hoc test was used for multiple comparisons between groups. And for two samples comparison, unpaired two-tailed Student’s *t*-test or paired two-tailed Student’s *t*-test was used for analysis. The Kruskal-Wallis test or Mann-Whitney *U* test was used to analyze the data which did not conform to the normality test and the homogeneity of variance test. Statistical analyses were performed using SPSS 15.0. *P* < 0.05 was considered a statistically significant difference.

## Results

### Generation of Specific Knockdown Transgenic Mice of *Pcp2*^*cre*^*;Ucp4*^*fl/fl*^ Mice

**Fig. 1:**
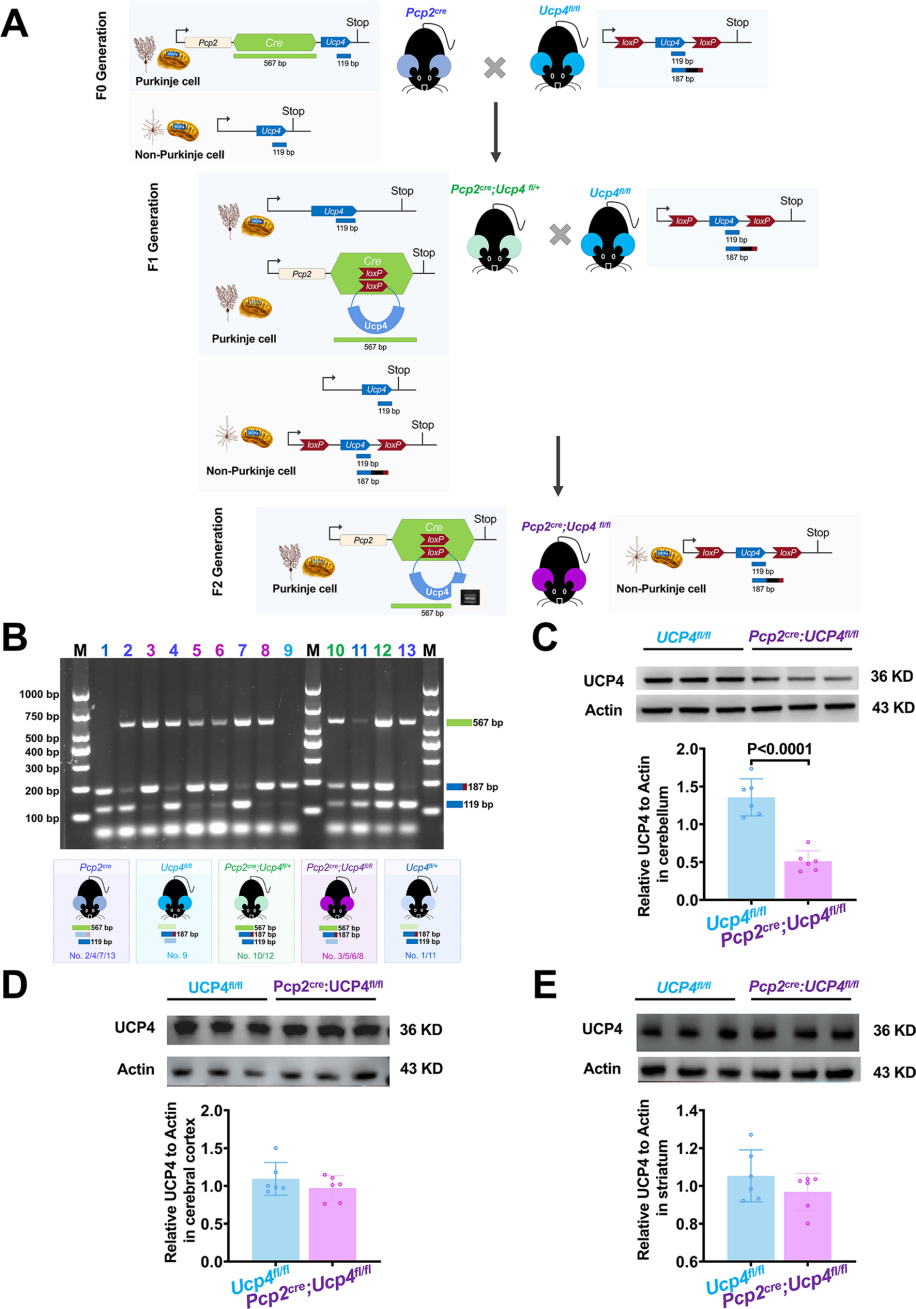
Conditional ablation of UCP4 within cerebellar PCs in *Pcp2*^*cre*^*;Ucp4*^*fl/fl*^ mice. (**A**) Strategy to generate conditional ablation of *Ucp4* within PCs in *Pcp2*^*cre*^*;Ucp4*^*fl/fl*^ mice by breeding *Ucp4*^*fl/fl*^ mice with *Pcp2*^*cre*^ mice. *Pcp2*^*cre*^ mice and *Ucp4*^*fl/fl*^ mice were used in the P0 generation, and generated *Pcp2*^*cre*^*;Ucp4*^*fl/+*^ mice as the F1 generation. *Pcp2*^*cre*^ mice had a *Cre* recombinase sequence inserted at the 3′ end of the *Pcp2* allele, which targeted to cerebellar PCs but not non-Purkinje cells. In the *Ucp4*^*fl/fl*^ mice, loxP sites were buried in the terminal end of the *Ucp4* sequence across all cells, including cerebellar PCs and non-Purkinje cells. F1 *Pcp2*^*cre*^*;Ucp4*^*fl/+*^ mice expressed *Cre* recombinase exclusively from one parental allele in PCs to cut the *Ucp4* loxp sequence. Then, crossing *Pcp2*^*cre*^*;Ucp4*^*fl/+*^ mice and *Ucp4*^*fl/fl*^ mice, *Pcp2*^*cre*^*;Ucp4*^*fl/fl*^ mice were generated as the F2 generation, in which almost all PCs lost the *Ucp4* sequence attributed to the cutting of loxP sites by *Cre* recombinase. (**B**) Representative PCR results of all transgenic mice. *Pcp2*^*cre*^ mice showed two bands of 567 bp and 119 bp as No. 2, 4, 7, and 13. *Ucp4*^*fl/fl*^ mice showed one band of 187 bp as No. 9. *Pcp2*^*cre*^*;Ucp4*^*fl/+*^ mice showed three bands of 567 bp, 187 bp, and 119 bp as No. 10 and 12. *Ucp4*^*fl/+*^ mice showed two bands of 187 bp and 119 bp as Nos. 1 and 11. *Pcp2*^*cre*^*;Ucp4*^*fl/fl*^ mice showed two bands of 567 bp and 187 bp as Nos. 3, 5, 6, and 8. (**C**) Representative image and quantification Western blot results of UCP4 in cerebellum. (**D**) Representative image and quantification Western blot results of UCP4 in cerebral cortex. (**E**) Representative image and quantification Western blot results of UCP4 in striatum. The data were analyzed by unpaired *t*-test. The data are shown as the mean ± SD; *n* = 6 mice per group. *P* < 0.05 was considered a statistically significant difference

Figure 1A shows the strategy employed to generate conditional ablation of *Ucp4* within PCs in *Pcp2*^*cre*^*;Ucp4*^*fl/fl*^ mice by breeding *Ucp4*^*fl/fl*^ mice with *Pcp2*^*cre*^ mice. In the P0 generation, *Pcp2*^*cre*^ mice and *Ucp4*^*fl/fl*^ mice were used. After crossing *Pcp2*^*cre*^ mice and *Ucp4*^*fl/fl*^ mice, *Pcp2*^*cre*^*;Ucp4*^*fl/+*^ mice were generated as the F1 generation. Then after crossing *Pcp2*^*cre*^*;Ucp4*^*fl/+*^ mice with *Ucp4*^*fl/fl*^ mice, *Pcp2*^*cre*^*;Ucp4*^*fl/fl*^ mice were generated as the F2 generation. In *Pcp2*^*cre*^*;Ucp4*^*fl/fl*^ mice, almost all of the PCs lost the *Ucp4* sequence, which was attributed to *Cre* recombinase cutting *loxP* sites, while non-PCs expressed the *Ucp4* sequence with a *loxP* site. These experiments demonstrated that *Ucp4* was specifically deleted in cerebellar PCs in *Pcp2*^*cre*^*;Ucp4*^*fl/fl*^ mice.

Genotype was confirmed by polymerase chain reaction (PCR) (Fig. 1B). There were five genotypes of mice in this study: (1) *Pcp2*^*cre*^ mice (Nos. 2, 4, 7, and 13); (2) *Ucp4*^*fl/fl*^ mice (No. 9); (3) *Pcp2*^*cre*^*;Ucp4*^*fl/+*^ mice (Nos. 10 and 12); (4) *Pcp2*^*cre*^*;Ucp4*^*fl/fl*^ mice (Nos. 3, 5, 6, and 8); and (5) *Ucp4*^*fl/+*^ mice (Nos. 1 and 11). In the following study, *Pcp2*^*cre*^*;Ucp4*^*fl/fl*^ mice were used as the experimental group, and *Ucp4*^*fl/fl*^ mice were used as control group.

### Confirmation of the Specific Knockdown of *Ucp4* in PCs by Western Blot, Double Immunofluorescent Staining, and Triple RNAscope In Situ Hybridization

The specific ablation of Ucp4 in PCs was confirmed by Western blot (Fig. 1C–E). The expression level of UCP4 relative to the internal reference Actin decreased by 62.42% in *Pcp2*^*cre*^*;Ucp4*^*fl/fl*^ mice, compared to *Ucp4*^*fl/fl*^ mice (Fig. 1C). Besides, there was no difference in both cerebral cortex and striatum between the two groups (Fig. 1D and E). Also, the results showed no significant expression difference of three other important mitochondrial factors among *Ucp4*^*fl/fl*^ mice, *Pcp2*^*cre*^*;Ucp4*^*fl/+*^ mice, and *Pcp2*^*cre*^*;Ucp4*^*fl/fl*^ mice, DRP1 (*P* = 0.1188), OPA1 (*P* = 0.5837), and MFN2 (*P* = 0.6413) (Fig. S2). These results confirmed the conditional ablation of *Ucp4* exclusively in cerebellar PCs.

**Fig. 2:**
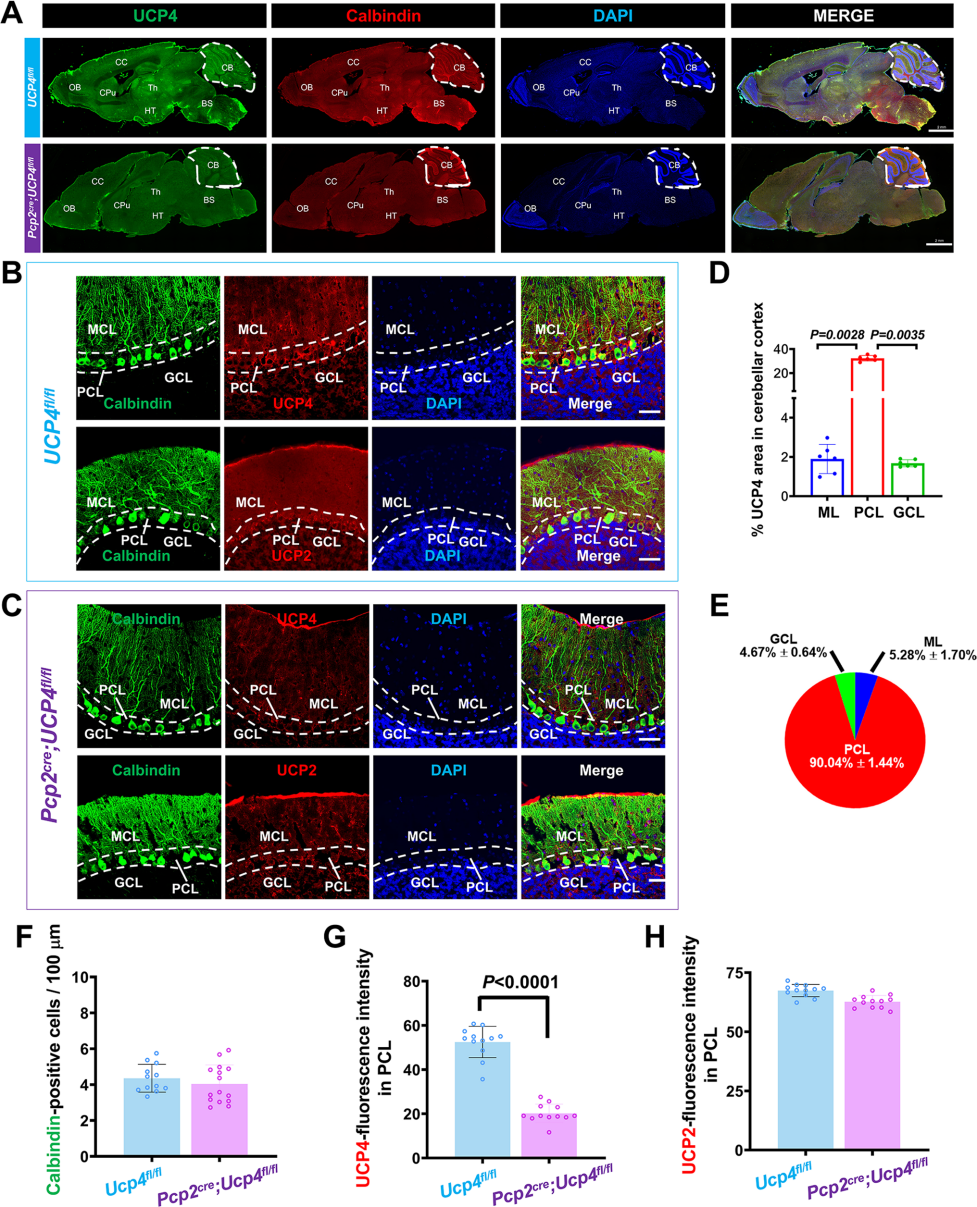
Double immunofluorescent staining confirmed the specific ablation of *Ucp4* in PCs of *Pcp2*^*cre*^*;Ucp4*^*fl/fl*^ mice. (**A**) Confocal images of UCP4 expression in total sagittal brain sections of *Ucp4*^*fl/fl*^ mice (top line) and *Pcp2*^*cre*^*;Ucp4*^*fl/fl*^ mice (bottom line). All confocal images in the top panel show the co-labeling of calbindin (a marker of Purkinje cells, green), UCP4 protein (red), and DAPI (blue). All confocal images in the bottom panel show the co-labeling of calbindin, UCP2 protein (red), and DAPI (blue). Bars = 2 mm. (**B** and **C**) Confocal images of UCP4 expression in PCs of *Ucp4*^*fl/fl*^ mice (B) and *Pcp2*^*cre*^*;Ucp4*^*fl/fl*^ mice (**C**). All confocal images in the top panel show the co-labeling of calbindin (a marker of Purkinje cells, green), UCP4 protein (red), and DAPI (blue). All confocal images in the bottom panel show the co-labeling of calbindin, UCP2 protein (red), and DAPI (blue). Bars = 50 μm. (**D**) Percent of the absolute value of UCP4-fluorescence in three layers of ML, PCL, and PCL to in the cerebellum in the control *Ucp4*^*fl/fl*^ mice. (**E**) Pie graph showing the ratio of UCP4 in three layers of ML, PCL, and PCL in the cerebellum in the control *Ucp4*^*fl/fl*^ mice. (**F**) Quantification of calbindin-positive PCs. (**G**) Quantification of UCP4-fluorescence intensity in PCL. (H) Quantification of UCP2-fluorescence intensity in the PCL. The data are shown as the mean ± SD. These results were analyzed by unpaired *t*-test and one-way ANOVA and LSD post hoc test or Kruskal-Wallis test, *n* = 6 mice per group. *P* < 0.05 was considered a statistically significant difference. BS, brain stem; CB, cerebellum; CC, cerebral cortex; Cpu, caudate putamen; GCL, granular cell layer; HT, hypothalamus; ML, molecular layer; OB, olfactory brain; PCL, Purkinje cell layer; Th, thalamus

The double immunofluorescent staining (Fig. 2A) showed the distributions of UCP4-positive immunostainings (red) and calbindin-positive PCs (green) in the whole brain at the middle sagittal sections. UCP4-positive immunostainings were expressed in most brain areas. Among them, UCP4 is highly expressed in following brain regions, including the olfactory brain (OB), cerebral cortex (CC), caudate putamen (Cpu), thalamus (Th), hypothalamus (HT), brain stem (BS), and cerebellum (CB), which was consistent with previous reports. And calbindin-positive PCs were distributed in the PCL of cerebellar cortex.

We further focused on the detailed distributions of both UCP4- and calbindin-positive immunostainings within the cerebellum of *Ucp4*^*fl/fl*^ mice (Fig. 2B), and *Pcp2*^*cre*^*;Ucp4*^*fl/fl*^ mice (Fig. 2C). We first calculated the percentages of UCP4 intensity in three layers of molecular layer (ML), Purkinje cell layer (PCL), and granular cell layer (GCL) of cerebellar cortex in the control *Ucp4*^*fl/fl*^ mice. And the values showed that the percentage of UCP4-fluorescence in PCL was the most, when compared with that in ML and GCL (Fig. 2D). The graph in Fig. 2E further showed that the percentage of UCP4-fluorescence in PCL was about 90%, indicating that it was reasonable that the UCP4-fluorescence in PCL could be used as indicator to reflect the change of UCP4 expression level after *Ucp4* knockdown. The graph in Fig. 2F showed that there was no significant difference in calbindin-positive cells per 100 μm between the control *Ucp4*^*fl/fl*^ mice (4.64 ± 0.83) and the experimental *Pcp2*^*cre*^*;Ucp4*^*fl/fl*^ mice (4.68 ± 0.89) (*P* = 0.9970), indicating that the conditional ablation of *Ucp4* in PCs would not induced PCs loss. The graph in Fig. 2G demonstrated that the fluorescence intensity of UCP4 within the PCL decreased significantly by 61.53% in *Pcp2*^*cre*^*;Ucp4*^*fl/fl*^ mice, when compared to that in homozygous *Ucp4*^*fl/fl*^ mice (*P* < 0.0001). To rule out changes of UCP4 expression in the brain, we detected it in cerebral cortex (Fig. S3) and striatum (Fig. S4) using immunofluorescent staining. We found that there were no differences of UCP4 expressions in these two regions between *Ucp4*^*fl/fl*^ mice and *Pcp2*^*cre*^*;Ucp4*^*fl/fl*^ mice.

In order to study whether or not the specific ablation of *Ucp4* in PCs would affect the expression of UCP2, the analog of UCP4, the double immunofluorescent staining of UCP2, and calbindin were performed. The graph in Fig. 2H showed that there was no significant difference in UCP2-fluorescence intensity in PCL between the control *Ucp4*^*fl/fl*^ mice and the experimental *Pcp2*^*cre*^*;Ucp4*^*fl/fl*^ mice (*P* = 0.0177).

**Fig. 3:**
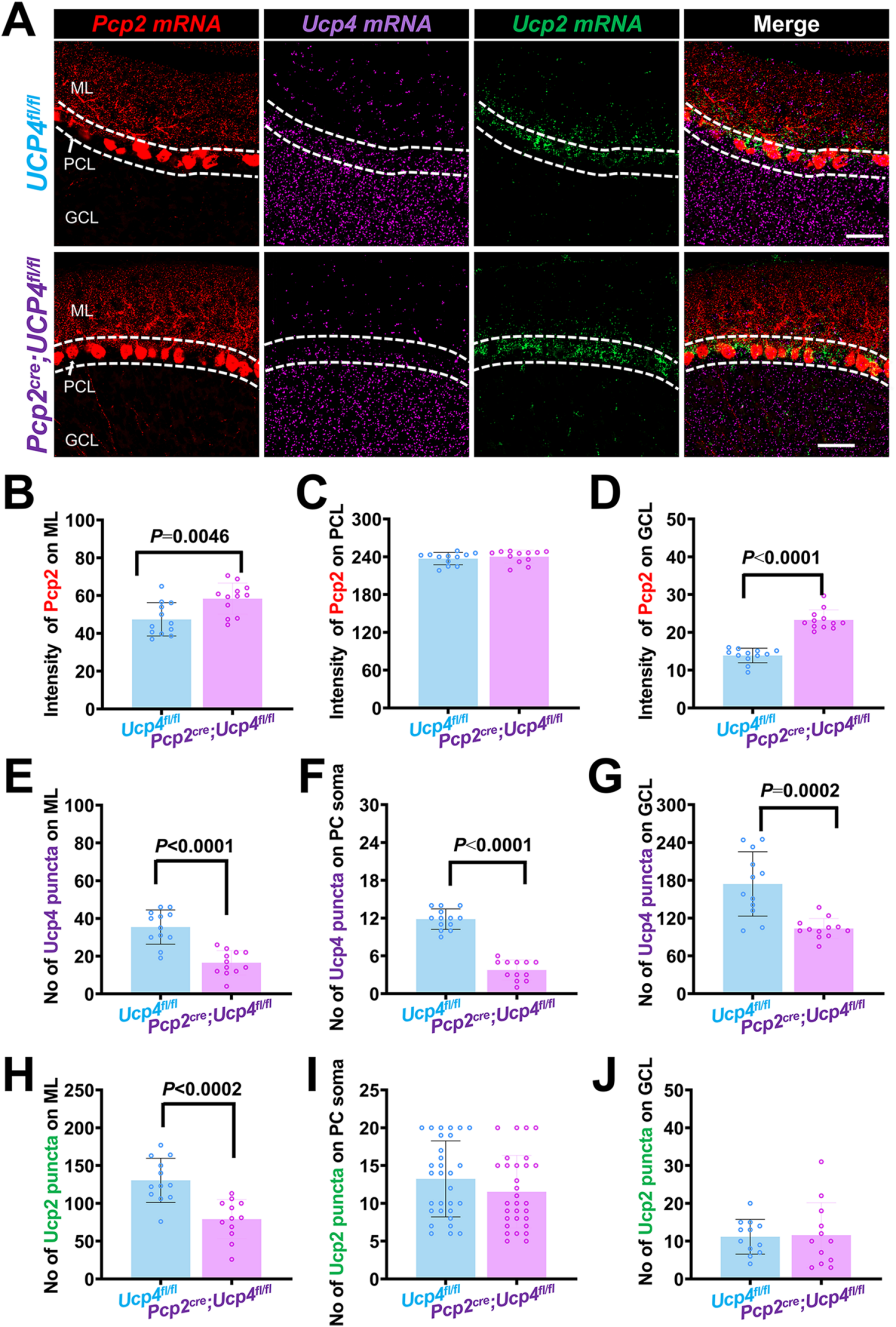
Triple RNAscope in situ hybridizations further confirmed the specific ablation of *Ucp4* in PCs of *Pcp2*^*cre*^*;Ucp4*^*fl/fl*^ mice. (**A**) Confocal images of co-labeling of *Pcp2* *mRNA* (a probe of Purkinje cells; red), *Ucp4* *mRNA* (violet), and *Ucp2* *mRNA* (green) in the cerebellar cortex of *Ucp4*^*fl/fl*^ mice (top panel) and *Pcp2*^*cre*^*;Ucp4*^*fl/fl*^ mice (bottom panel). Bars = 50 μm. (**B–D**) Quantification of *Pcp2* mRNA-positive intensities (red) in ML (B), PCL (C), and GCL (D), respectively. (**E–G**) Quantification of number of *Ucp4* mRNA-positive dots (violet) in ML (E), soma of PCs (F), and GCL (G), respectively. The number of *Ucp4* mRNA-positive dots in soma of PCs decreased approximately 70% in *Pcp2*^*cre*^*;Ucp4*^*fl/fl*^ mice when compared to homozygous *Ucp4*^*fl/fl*^ mice. (**H–J**) Quantification of number of *Ucp2* mRNA-positive dots (green) in ML (H), soma of PCs (I), and GCL (J), respectively. Statistical analysis was performed by unpaired *t*-test. The data are shown as the mean ± SD, *n* = 3 mice per group. *P* < 0.05 was considered a statistically significant difference. GCL, granular cell layer; ML, molecular layer; PCL, Purkinje cell layer

Triple RNAscope in situ hybridization results (Fig. 3A) were in general consistent with that by double immunofluorescent staining. It was shown that there was no significant difference in the number of *Pcp2* mRNA-dots (red) on both ML (Fig. 3B) and GCL (Fig. 3D), as well as in the intensities of *Pcp2* mRNA-dots on PCs soma (Fig. 3C), between *Ucp4*^*fl/fl*^ mice and *Pcp2*^*cre*^*;Ucp4*^*fl/fl*^ mice. And it was indicated that the number of *Ucp4* mRNA-dots (violet) on ML (Fig. 3E), or PCs soma (Fig. 3F), or GCL (Fig. 3G), decreased by approximately 53.42%, 68.35%, and 40.63% in *Pcp2*^*cre*^*;Ucp4*^*fl/fl*^ mice, respectively, when compared with those in *Ucp4*^*fl/fl*^ mice (*P* < 0.05). We noted that there was no significant difference in the number of *Ucp2* mRNA-dots (green) on both PCL (Fig. 3I) and GCL (Fig. 3J), between *Ucp4*^*fl/fl*^ mice and *Pcp2*^*cre*^*;Ucp4*^*fl/fl*^ mice, although the number of *Ucp2* mRNA-dots (green) on ML (Fig. 3H) decreased in *Pcp2*^*cre*^*;Ucp4*^*fl/fl*^ mice (*P* < 0.05).

Therefore, we confirmed the specific *Ucp4* deletion in the PCs of *Pcp2*^*cre*^*;Ucp4*^*fl/fl*^ mice.

### Bradykinesia Performance of *Pcp2*^*cre*^*;Ucp4*^*fl/fl*^ Mice by Combined Behavioral Tests

**Fig. 4:**
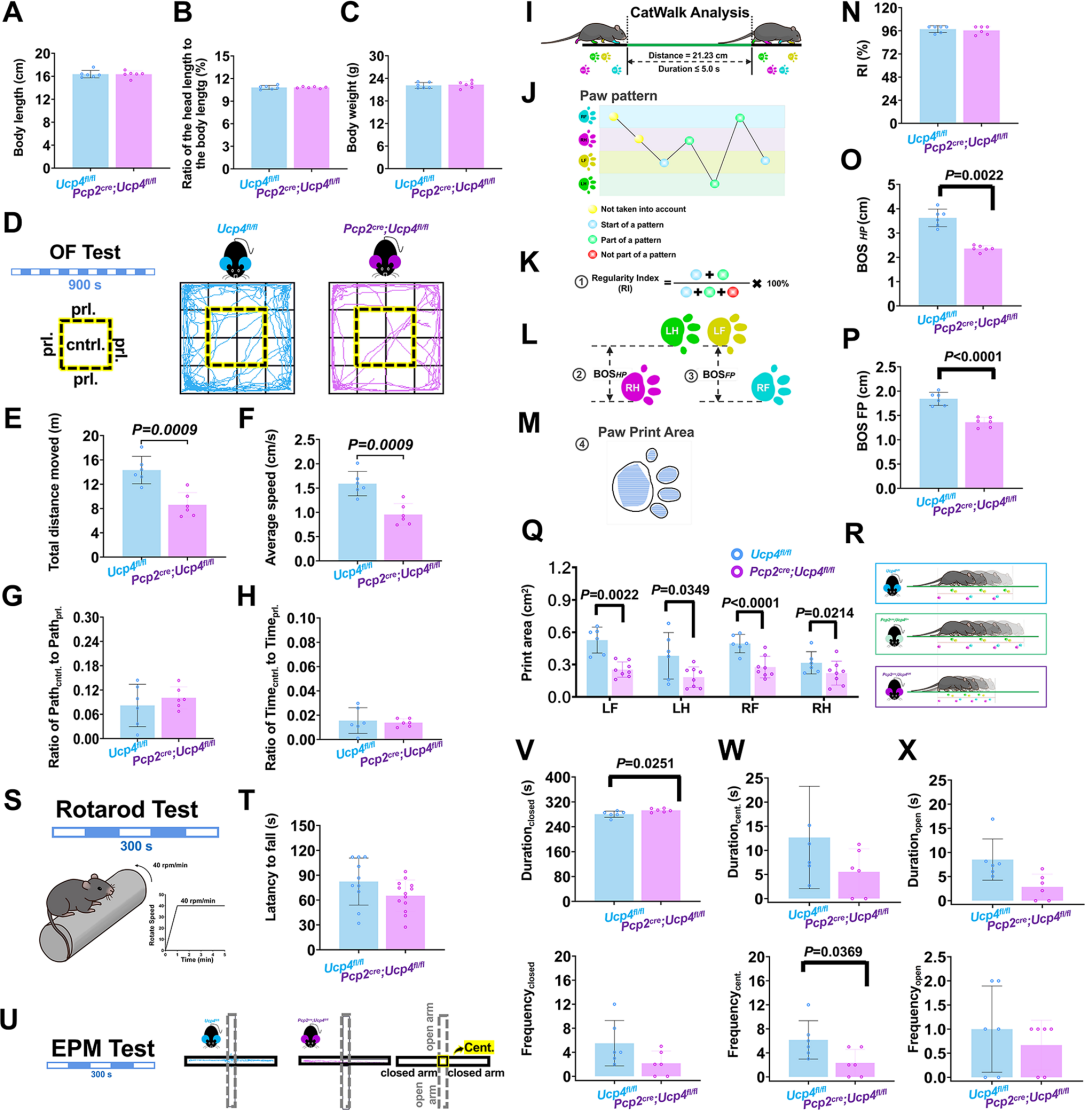
The combined behavioral tests of open field (OF), CatWalk, Rotarod, and elevated plus maze (EPM) showed the bradykinesia of *Pcp2*^*cre*^*;Ucp4*^*fl/fl*^ mice. (**A–C**) The average body length (A), the average ratio of the head length to the body length (B), and the average body weight (C) showed no significant difference between *Ucp4*^*fl/fl*^ mice (male, 8-weeks-old, *n* = 5) and *Pcp2*^*cre*^*;Ucp4*^*fl/fl*^ mice (male, 8-weeks-old, *n* = 6). (**D–H**) Representative traces (D) of the open field (OF) test and quantification of the total distance (E), average speed (F), the Ratio of Path_cent._ to Path_prl._ (G), and the Ratio of Time_cent._ to Time_prl._ (H) between the two groups. (**I–M**) Schematic of CatWalk analysis (I) and all paw patterns were recorded (J). The parameters of the regularity index (RI) (K), the base of support (BOS) (L) between either the hind paws (BOS_*HP*_) and the front paws (BOS_*FP*_), and the print area (M) of the left front paw (LF), the left hind paw (LH), the right front paw (RF), and the right hind paw (RH) were analyzed. (**N–R**) Quantification of the RI (N), BOS_*HP*_ (O), BOS_*FP*_. (P), all paw print areas (R), and the cartoon (S) showing the bradykinesia of *Pcp2*^*cre*^*;Ucp4*^*fl/fl*^ mice. (**S** and **T**) Schematic of Rotarod test (S) and quantification of the value of the latency to fall (T). (**U–X**) Representative traces of elevated plus maze (EPM) test (U), and quantification of Duration_closed_ and Frequency_closed_) (V), Duration_cent._ and Frequency_cent._ (W), and Duration_open_ and Frequency_open_ (X). The data are shown as the mean ± SD. Statistical analysis was performed by unpaired *t*-test. *P* < 0.05 was considered a statistically significant difference. BOS_FP/HP_, base of support of front paws/hind paws; Duration_cent./closed/open_, duration in central platform/closed arms/open arms; EPM, elevated plus maze; Frequency_cent./closed/open_, frequency in central platform/closed arms/open arms; LF, left front paw; LH, left hind paw; OF, open field; Ratio of Path_cent._ to Path_prl._, ratio of the central distance moved to the periphery; Ratio of Time_cent._ to Time_prl._, ratio of time spent in the center to the periphery; RF, right front paw; RH, right hind paw

First, it was found that the physical appearance of 8-weeks-old male *Pcp2*^*cre*^*;Ucp4*^*fl/fl*^ mice was indistinguishable from the littermate of *Ucp4*^*fl/fl*^ mice (8-weeks-old male) and *Pcp2*^*cre*^*;Ucp4*^*fl/+*^ mice (8-weeks-old male). They had the similar average body length of 16.37 ± 0.38 cm (measured from head to tail end) (Fig. 4A), similar average ratio of the head length (defined as the distance from the tip of the nose to the imaginary line between the ears) to the body length of 10.92% ± 0.81% (Fig. 4B), and similar average body weight of 22.42 ± 0.83 g (Fig. 4C). And they had normal black body hair and pupils (data not shown).

To explore the locomotor capacity of *Pcp2*^*cre*^*;Ucp4*^*fl/fl*^ mice, we performed OF test with a duration of 900 s for a long autonomic movement appraisal (Fig. 4D–H). Figure 4E shows that the total distance moved was reduced by 40.0% from 14.32 ± 2.25 m in *Ucp4*^*fl/fl*^ mice to 8.60 ± 2.01 m in *Pcp2*^*cre*^*;Ucp4*^*fl/fl*^ mice (*P* = 0.0093). Figure 4F shows that the average speed was reduced by 39.6% from 1.59 ± 0.25 cm/s in *Ucp4*^*fl/fl*^ mice to 0.96 ± 0.22 cm/s in *Pcp2*^*cre*^*;Ucp4*^*fl/fl*^ mice (*P* = 0.0093). Figure 4G and H shows that there was no significant difference in the ratio of Path_cent_. to Path_prl_. or ratio of Time_cent_. to Time_prl_. between that of *Ucp4*^*fl/fl*^ mice and that of *Pcp2*^*cre*^*;Ucp4*^*fl/fl*^ mice. These results reflected the bradykinesia of *Pcp2*^*cre*^*;Ucp4*^*fl/fl*^ mice.

We also observed that in the open field test (Fig. S5A), there was no difference among three mice groups of *Ucp4*^*fl/fl*^ mice, *Pcp2*^*cre*^*;Ucp4*^*fl/+*^ mice, and *Pcp2*^*cre*^*;Ucp4*^*fl/fl*^ mice, whenever in total distance moved (Fig. S5B), average speed (Fig. S5C), ratio of Path_contrl._ to Path_prl_ (Fig. S5D)_._, and ratio of Time_contrl._ to Time_prl._ (Fig. S5E). These results confirmed it was reasonable to use *Ucp4*^*fl/fl*^ mice as the control in the whole studies.

To explore the stepping alterations of *Pcp2*^*cre*^*;Ucp4*^*fl/fl*^ mice, we next analyzed CatWalk data (Fig. 4I–M). Figure 4N shows that there was no significant difference in RI between *Ucp4*^*fl/fl*^ mice and *Pcp2*^*cre*^*;Ucp4*^*fl/fl*^ mice (*P* = 0.8436), confirming that *Pcp2*^*cre*^*;Ucp4*^*fl/fl*^ mice had normal inter-paw coordination. Figure 4O and P shows that the BOS_*HP*_ was reduced by 34.8% from 3.62 ± 0.36 cm in *Ucp4*^*fl/fl*^ mice to 2.36 ± 0.12 m in *Pcp2*^*cre*^*;Ucp4*^*fl/fl*^ mice (*P* = 0.0006), and the BOS_*FP*_ was reduced by 26.1% from 1.84 ± 0.13 cm in *Ucp4*^*fl/fl*^ mice to 1.36 ± 0.10 cm in *Pcp2*^*cre*^*;Ucp4*^*fl/fl*^ mice (*P <* 0.0001). These results demonstrated the reduced amplitude of stepping of *Pcp2*^*cre*^*;Ucp4*^*fl/fl*^ mice. Figure 4Q shows that all of the paw print areas presented a considerable decline (approximately 58.1% for LF, 60.4% for LH, 54.1% for RF, and 44.7% for RH) in *Pcp2*^*cre*^*;Ucp4*^*fl/fl*^ mice. These results confirmed the bradykinesia of *Pcp2*^*cre*^*;Ucp4*^*fl/fl*^ mice (Fig. 4R).

Rotarod test (Figs. 4S and 4T) confirmed the absence of ataxia in *Pcp2*^*cre*^*;Ucp4*^*fl/fl*^ mice because there was no significant difference of the latency to fall between that in *Ucp4*^*fl/fl*^ mice and in *Pcp2*^*cre*^*;Ucp4*^*fl/fl*^ mice (*P* = 0.2222).

Considering PCs are related to emotional performance [35], we next employed EPM test (Fig. 4U–X). There were no significant differences in Duration_closed_ and Frequency_closed_ (Fig. 4V), Duration_cent._ and Frequency_cent._ (Fig. 4W), and Duration_open_ and Frequency_open_ (Fig. 4X), among three groups, which indicated that the deletion of UCP4 in PCs did not greatly affect animal emotion.

### No Changes of the Electromyogram Recordings in *Pcp2*^*cre*^*;Ucp4*^*fl/fl*^ Mice

**Fig. 5:**
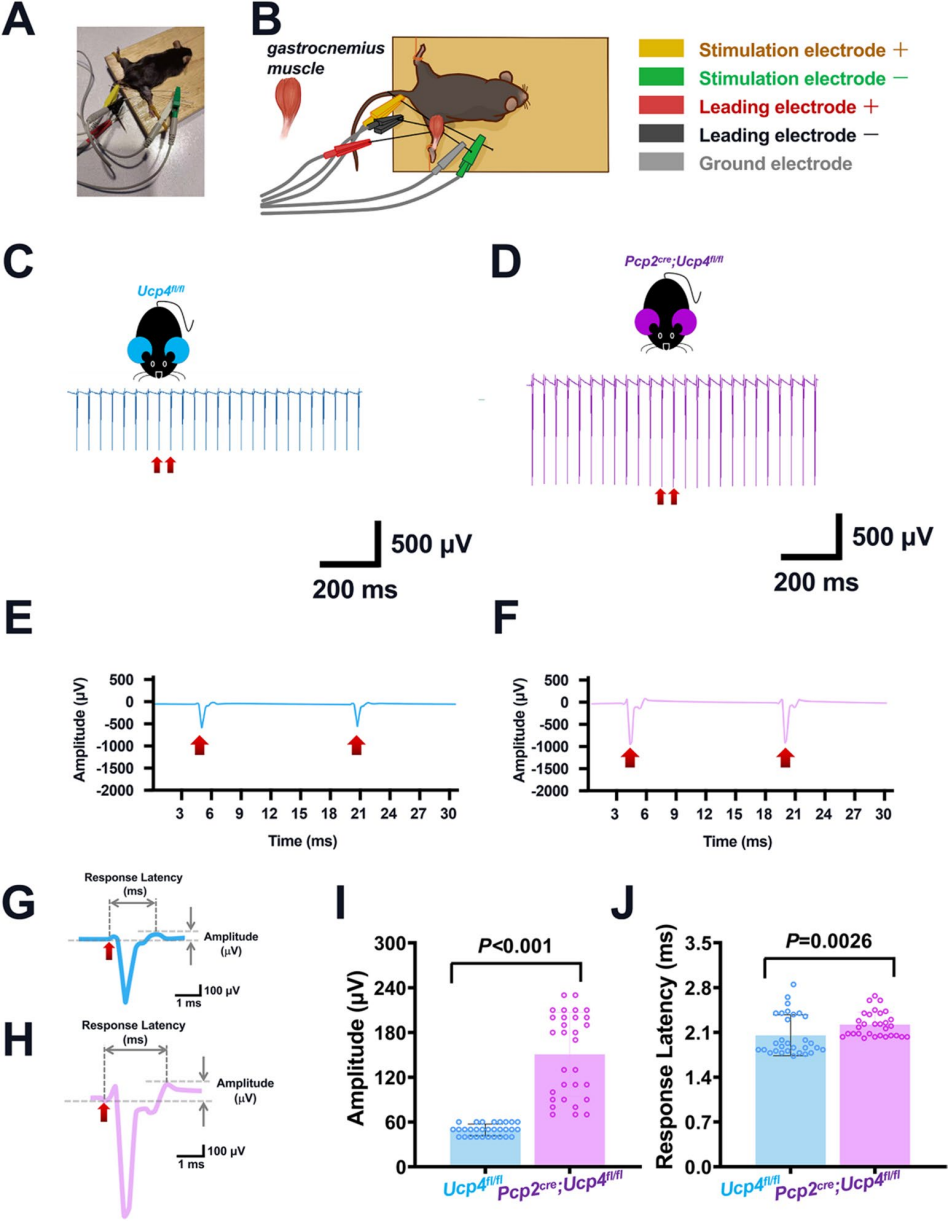
Electromyogram (EMG) recordings indicated an absence of muscle damage of *Pcp2*^*cre*^*;Ucp4*^*fl/fl*^ mice. (**A**) Representing photograph showing the EMG recording of the mouse gastrocnemius muscle. (**B**) Schematic of EMG performed on the gastrocnemius muscle. The yellow probe indicates the positive (+) stimulation electrode; the green probe indicates the negative (−) stimulation electrode; the red probe indicates the positive (+) leading electrode; the black probe indicates the negative (−) leading electrode; the gray probe indicates the ground electrode. (**C–F**) Representative traces of EMG from *Ucp4*^*fl/fl*^ (C) and *Pcp2*^*cre*^*;Ucp4*^*fl/fl*^ mice (D). Two representative spikes marked by red arrows are magnified in (E) and (F), correspondingly. (**G** and **H**) A representative spike from *Ucp4*^*fl/fl*^ mice (G) and *Pcp2*^*cre*^*;Ucp4*^*fl/fl*^ mice (H). (**I** and **J**) Quantification of amplitude (I) and response latency (J) of EMG. Statistical analysis was performed by unpaired *t*-test. The data are shown as the mean ± SD, *n* = 3 (male, 8-weeks-old) mice per group. *P* < 0.05 was considered a statistically significant difference. EMG, electromyogram

In the next, whether or not the hypotonia was present in *Pcp2*^*cre*^*;Ucp4*^*fl/fl*^ mice was studied by electromyogram recordings (EMG) detection on the gastrocnemius muscle (Fig. 5A and B). EMG tracings of *Ucp4*^*fl/fl*^ mice (Fig. 5C) and *Pcp2*^*cre*^*;Ucp4*^*fl/fl*^ mice (Fig. 5D) were shown. Their corresponding typical firing recordings were amplified in Fig. 5E–H and further calculated shown in Fig. 5G and H. Figure 5I and J shows that the *Pcp2*^*cre*^*;Ucp4*^*fl/fl*^ mice had a significantly higher amplitude (150.67 ± 56.14 μV, *P* < 0.0001) and response latency (2.22 ± 0.19 ms, *P* = 0.0146) than the control *Ucp4*^*fl/fl*^ mice (49.33 ± 7.85 μV and 2.05 ± 0.32 ms, respectively). The present EMG results excluded the muscle damage in *Pcp2*^*cre*^*;Ucp4*^*fl/fl*^ mice; hence in the following patch clamp recording, we focused on the changes of the electrical properties of PCs.

### Altered Spontaneous and Evoked Firings Properties of PCs in *Pcp2*^*cre*^*;Ucp4*^*fl/fl*^ Mice by Electrical Patch Clamp Recordings

**Fig. 6:**
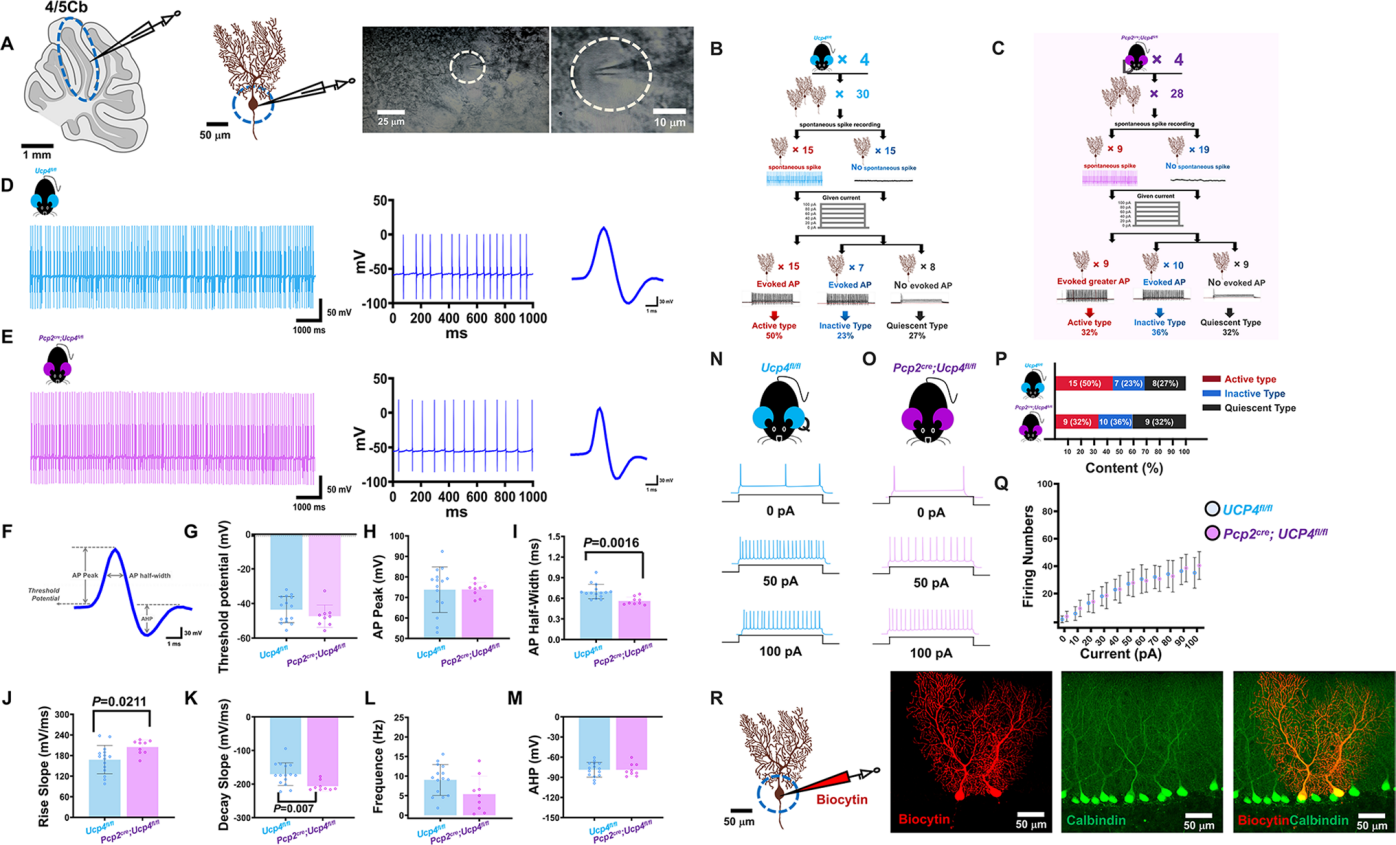
Electrical patch clamp recordings showing the changes of both spontaneous and evoked firing properties of PCs in *Pcp2*^*cre*^*;Ucp4*^*fl/fl*^ mice. (**A**) Schematic showing the patch clamp recordings on 4 and 5 lobes of the cerebellar cortex (4/5 Cb). Bar = 1 mm, 50 μm, 25 μm, and 10 μm, respectively. (**B** and **C**) Schematic showing the three types of PCs from *Ucp4*^*fl/fl*^ mice (B) and *Pcp2*^*cre*^*;Ucp4*^*fl/fl*^ mice (C). (**D** and **E**) Representative raw traces for 1 minute recording showing the spontaneous spikes of PCs, from *Ucp4*^*fl/fl*^ mice (D) and *Pcp2*^*cre*^*;Ucp4*^*fl/fl*^ mice (E). (**F**) Seven parameters were measured. (**G–M**) Quantification of threshold potential (mV) (G), the action potential (AP) peak (mV) (H), the half-width of AP (ms) (I), the rise slope (mV/ms) (J) and decay slope (mV/ms) (K), the frequency (Hz) (L), and the afterhyperpolarization (AHP, mV) (M). (**N** and **O**) Representative evoked spikes by current stimulation in *Ucp4*^*fl/fl*^ mice (N) and *Pcp2*^*cre*^*;Ucp4*^*fl/fl*^ mice (O). (**P**) Proportion of firing patterns. The red percentage represents active type; blue percentage represents inactive type; and black percentage represents quiescent type. (**Q**) Quantification of firing numbers. (**R**) Schematic confirming the location of the patch clamp on the soma of PCs by micro injection of biocytin and the representative confocal photograph of PCs immunostained by both red biocytin and green calbindin. Statistical analysis was performed by unpaired *t*-test. The data are shown as the mean ± SD, *n* = 4 mice per group. *P* < 0.05 was considered a statistically significant difference

Next, the spontaneous or evoked firings properties of PCs of *Pcp2*^*cre*^*;Ucp4*^*fl/fl*^ mice were studied by the electrical patch clamp recordings on freshly isolated 4/5 lobes of the cerebellum (4/5Cb). Figure 6A shows the insertion of the glass electrode into the soma of PCs. We totally recorded 30 PCs in *Ucp4*^*fl/fl*^ mice (Fig. 6B) and 28 PCs in *Pcp2*^*cre*^*;Ucp4*^*fl/fl*^ mice (Fig. 6C). We found 15 of the total 30 PCs in *Ucp4*^*fl/fl*^ mice and 9 of 28 in *Pcp2*^*cre*^*;Ucp4*^*fl/fl*^ mice had spontaneous firings, respectively. We then analyzed the spontaneous firing patterns of PCs in *Ucp4*^*fl/fl*^ mice (Fig. 6D) and in *Pcp2*^*cre*^*;Ucp4*^*fl/fl*^ mice (Fig. 6E). As shown in Fig. 6F–M, 7 parameters were measured. It was found that the AP half-width of *Pcp2*^*cre*^*;Ucp4*^*fl/fl*^ mice was reduced by 19.7% compared to that of *Ucp4*^*fl/fl*^ mice (*Ucp4*^*fl/fl*^ mice: 0.70 ± 0.11 ms; *Pcp2*^*cre*^*;Ucp4*^*fl/fl*^ mice: 0.56 ± 0.06 ms; *P* = 0.0049) (Fig. 6I); and the rise and decay slope became steeper by 21.8% and 21.1%, respectively, after *Ucp4* conditional ablation (rise slope: 168.05 ± 41.06 mV/ms in *Ucp4*^*fl/fl*^ mice and 204.72 ± 20.62 mV/ms in *Pcp2*^*cre*^*;Ucp4*^*fl/fl*^ mice, *P* = 0.0467, Fig. 6J; decay slope: −170.52 ± 33.70 mV/ms in *Ucp4*^*fl/fl*^ mice and −206.41 ± 14.55 mV/ms in *Pcp2*^*cre*^*;Ucp4*^*fl/fl*^ mice, *P* = 0.0105, Fig. 6K).

Then, the evoked spikes of PCs in *Ucp4*^*fl/fl*^ mice (Fig. 6N) and P*cp2*^*cre*^*;Ucp4*^*fl/fl*^ mice (Fig. 6O) were analyzed. Figure 6P shows that the proportion of both active and inactive PCs (excitable) reduced from 73% in *Ucp4*^*fl/fl*^ mice to 68% in *Pcp2*^*cre*^*;Ucp4*^*fl/fl*^ mice. Figure 6Q shows that there was no significant difference in the total firing number of both active and inactive PCs at every given current between any two groups. Finally, the biocytin (red) injection confirmed the recorded cells were PCs (Fig. 6R).

Consequently, we speculated that the excitability of PCs was reduced by specific ablation of *Ucp4*.

### Altered Mitochondrial Morphology and Mitochondrial Functions in Cerebellum of *Pcp2*^*cre*^*;Ucp4*^*fl/fl*^ Mice

**Fig. 7:**
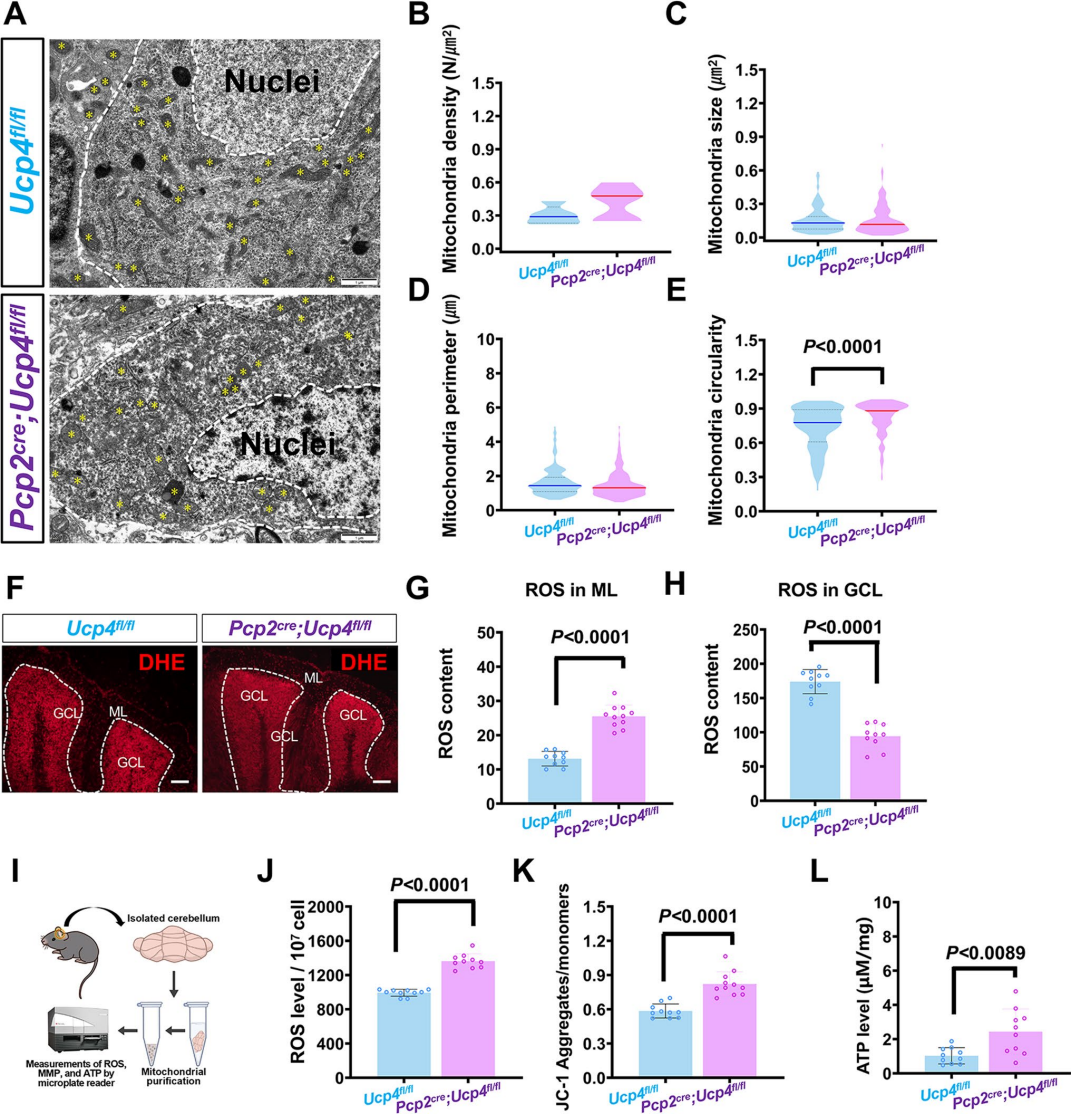
Mitochondrial morphology and function changed in cerebellum of *Pcp2*^*cre*^*;Ucp4*^*fl/fl*^. (**A**) Transmission electron microscope (TEM) images showing mitochondrial morphology (indicated by yellow star symbols) of cerebellum in *Ucp4*^*fl/fl*^ mice and *Pcp2*^*cre*^*;Ucp4*^*fl/fl*^ mice. (**B–E**) Quantification of mitochondrial density (B), area (C), perimeter (D) under TEM. (**F**) Confocal images of ROS generation in the cerebellum tissues from *Ucp4*^*fl/fl*^ mice and *Pcp2*^*cre*^*;Ucp4*^*fl/fl*^ mice. (**G**) Quantification of ROS in molecular layer (ML) of the cerebellum tissues, which was full of the dendritic trees of Purkinje cells, from *Ucp4*^*fl/fl*^ mice and *Pcp2*^*cre*^*;Ucp4*^*fl/fl*^ mice. (**H**) Quantification of ROS in granule cell layer (GCL) of the cerebellum tissues from *Ucp4*^*fl/fl*^ mice and *Pcp2*^*cre*^*;Ucp4*^*fl/fl*^ mice. (**I**) Flow chart of quantification of ROS, MMP and ATP. (**J–L**) Results of ROS (J), JC-1 (K), and ATP (L) of cerebellum. The data were analyzed by unpaired *t*-test. The data are shown as the mean ± SD; *n* = 3 mice per group. *P* < 0.05 was considered a statistically significant difference. ATP, adenosine triphosphate; MMP, mitochondrial membrane potential; ROS, reactive oxygen species

To observe the changes of mitochondrial morphology after *Ucp4* knockdown, the mitochondria within Purkinje cells were observed under transmission electron microscopy (TEM) (Fig. 7A). Furthermore, the analysis of mitochondrial density (Fig. 7B), mitochondrial area (Fig. 7C), and mitochondrial perimeter (Fig. 7D) in both control *Ucp4*^*fl/fl*^ mice and *Pcp2*^*cre*^*;Ucp4*^*fl/fl*^ mice was performed. *Pcp2*^*cre*^*;Ucp4*^*fl/fl*^ mice (Fig. 7A). The results showed that there was no significant difference in after *Ucp4* knockdown; however, the mitochondrial circularity (Fig. 7E) increased significantly. Reactive oxygen species (ROS) generation results (Fig. 7F–H) showed the significant increase of ROS in ML of the cerebellum tissues, which was full of the dendritic trees of Purkinje cells, in *Pcp2*^*cre*^*;Ucp4*^*fl/fl*^ mice, when compared to that of *Ucp4*^*fl/fl*^ mice. But there was no difference of ROS in GCL, which was full of granular cells, between the two groups. To further confirm the mitochondria function of cerebellum after *Ucp4* knockdown, ROS, JC-1, and ATP analyses were performed using microplate reader. The results showed that the knockdown of *Ucp4 *significantly increased ROS generation approximately by 27.08%, MMP level by 28.86%, and ATP level by 57.86% in the cerebellum, respectively (Fig. 7I–K). These results suggested that *Ucp4* knockdown affected mitochondrial morphology and functions in cerebellum of *Pcp2*^*cre*^*;Ucp4*^*fl/fl*^ mice.

## Discussion

To evaluate the role of *Ucp4* in Purkinje cells (PCs), we generated conditional deletion of *Ucp4* in PCs (*Pcp2*^*cre*^*;Ucp4*^*fl/fl*^ mice) by breeding *Ucp4*^*fl/fl*^ mice with *Pcp2*^*cre*^ mice. PCR, Western blot, double immunofluorescent staining, and triple RNAscope in situ hybridization confirmed the specific ablation of *Ucp4* in PCs in *Pcp2*^*cre*^*;Ucp4*^*fl/fl*^ mice. Open field test, CatWalk analysis, rotarod, and elevated plus maze tests showed that *Pcp2*^*cre*^*;Ucp4*^*fl/fl*^ mice displayed bradykinesia. The electromyogram recordings excluded the hypotonia in *Pcp2*^*cre*^*;Ucp4*^*fl/fl*^ mice. And the electrical patch clamp recordings showed the altered properties of both spontaneous and evoked firings in PCs of *Pcp2*^*cre*^*;Ucp4*^*fl/fl*^ mice. Also, the knockdown of Ucp4 significantly increased ATP, MMP, and ROS in the cerebellum. It was the first time to report a close relationship between UCP4 deletion with PCs impairment.

Previous studies have demonstrated that the mutation of many mitochondrial genes could lead to ataxia movement disorder. Aoki et al. [36] have reported that spinocerebellar ataxia type 31 (SCA31), an autosomal-dominant neurodegenerative disorder characterized by progressive cerebellar ataxia and PCs loss, is caused by the mutation of gene of an essential mitochondrial thymidine kinase 2 (TK2). Rumyantseva et al. [37] have found that the conditional PCs-specific deletion of mitochondrial aspartyl-tRNA synthetase (DARS2) causes a massive loss of PCs and ataxia. Previous reports [38–40] have found that the conditional deletion of AFG3L2 which encodes one of the subunits of the m-AAA protease in Bergmann glia cell leads to PCs degeneration and ataxia. And it has been shown that the m-AAA protease is important for mitochondrial homeostasis. Nair et al. [41] have investigated that the conditional PCs-specific deletion of mitochondrial fatty acid synthesis (mtFAS) causes a massive PCs loss and ataxia. SLC25A46 plays an important role in mitochondrial dynamics by mediating mitochondrial fission. Li et al. [42] have described that the Slc25a46 knock-out mouse has displayed severe ataxia which is mainly caused by degeneration of PCs. Combined with the present results, there is no doubt that the mitochondrial quality control is closely related to PCs homeostasis and motor function.

Mitochondria, as the main energy supplying organelles, provide the nervous system with ATP, which is necessary for survival. PCs function as the sole efferent neurons in the cerebellar cortex [43], which determines the highly dependent nature of PCs on mitochondria. UCPs are part of a superfamily of mitochondrial anionic carriers, which are located in the inner mitochondrial membrane (IMM) and reduce the proton-electrochemical gradient [5, 44]. Given the limited number of studies on UCP4 in CNS, the role of *Ucp4* in PCs is still unclear. Therefore, we crossed the *Pcp2*^*cre*^ mice with *Ucp4*^*fl/fl*^ mice to generate the specific *Ucp4* ablation mice. To our surprise, *Pcp2*^*cre*^*;Ucp4*^*fl/fl*^ mice maintained normal morphological features of PCs, had no ataxia, and presented a normal body appearance and balance ability (reflected by rotarod test). Importantly, these specific *Ucp4* ablation mice presented bradykinesia. Bradykinesia was first proposed by John Parkinson, who described it as a characteristic of slowness when performing movement [45], and it is now considered the most important motor defect of PD. In addition to the behavior test, the results of patch clamp and EMG suggested against muscle damage in our *Ucp4*-specific ablation mice and confirmed the firing changes in PCs. Consequently, we speculated that bradykinesia caused by *Ucp4*-specific ablation was related to the reduced proportion of PCs with spontaneous spikes.

UCP4 has been shown to reduce the MMP and ROS. The knockdown of UCP4 is reported to increase the MMP, while the over-expression of UCP4 is reported to reduce the MMP, ROS, and ATP [6–8]. Our present results are consistent with previous data that we have found the significant increases of ATP level, MMP, and ROS generation in cerebellum of *Pcp2*^*cre*^*;Ucp4*^*fl/fl*^. Thereafter, UCP4 might be a therapeutic target for the cerebellar-related movement disorder. The present study is the first to report a close relationship between UCP4 deletion with PCs impairment, and suggests the importance of UCP4 in the substantial support of mitochondrial function homeostasis in bradykinesia.

Although we detected the cerebellar mitochondrial function changes induced by the deletion of UCP4 in PCs, we did not define the exact mitochondrial alterations within the PCs. Therefore, it was necessary to explore the detailed mitochondrial functions in PCs after *Ucp4*-specific deletion.

## Conclusion

In conclusion, we generated *Pcp2*^*cre*^*;Ucp4*^*fl/fl*^ mice with the conditional knockdown of mitochondrial *Ucp4* in cerebellar PCs, and found these *Pcp2*^*cre*^*;Ucp4*^*fl/fl*^ mice displayed bradykinesia, which was possibly due to the reduced excitability of PCs induced by the oxidative crisis. The present study is the first to report a close relationship between UCP4 deletion with PCs impairment, and suggests the importance of UCP4 in the substantial support of mitochondrial function homeostasis in bradykinesia. We further deduce that UCP4 might be a therapeutic target for the cerebellar-related movement disorder.

### Supplementary information

ESM 1 (DOCX 4143 kb)

## Data Availability

The datasets used and analyzed in this study are available from Ya-Yun Wang upon reasonable request.

## Abbreviations

ACSF: Artificial cerebrospinal fluid
AD: Alzheimer’s disease
AHP: After hyperpolarization
AP: Action potential
BOS_FP/HP_: Base of support of front paws / hind paws
BS: Brain stem
CB: Cerebellum
CC: Cerebral cortex
Cpu: Caudate putamen
DCFH-DA: 2,7-Dichlorodi-hydrofluorescein diacetate
Drp1: Dynamin-related protein 1
Duration_cent./closed/open_: Duration in central platform/closed arms/open arms
EMG: Electromyogram
EPM: Elevated plus maze
FCCP: Farbonyl cyanide 4-(trifluoromethoxy) phenylhydrazone
*fl*: Flox
Frequency_cent./closed/open_: Frequency in central platform/closed arms/open arms
GCL: Granule cells layer
HT: Hypothalamus
IMM: Inner mitochondrial membrane
LF: Left front paw
LH: Left hind paw
ML: Molecular layer
Mcu: Mitochondrial calcium uniporter
Mfn1/2: Mitofusion 1/2
Mito Tempo: A mitochondria-targeted superoxide mimetic
ML: Molecular layer
MMP: Mitochondrial membrane potential
Nclx: Sodium/lithium/calcium exchanger
no: Number
OB: Olfactory brain
OF: Open field
OligoA: Ologomycin A
OMM: Outer mitochondrial membrane
OPA1: Optic atrophy 1
OXPHOS: Oxidative phosphorylation
PBS: Phosphate buffered saline
PBS-TX: Phosphate buffered saline containing 1% Triton X-100
PCL: Purkinje cell layer
Pcp2: Purkinje cell protein 2
PCR: Polymerase chain reaction
PCs: Purkinje cells
PD: Parkinson’s disease
PDK1: 3-Phosphoinositide-dependent protein kinase-1
PFA: Paraformaldehyde
Ratio of Path_cent._ to Path_prl._: Ratio of the central distance moved to the periphery
Ratio of Time_cent._ to Time_prl._: Ratio of time spent in the center to the periphery
RF: Right front paw
RH: Right hind paw
ROS: Reactive oxygen species
rpm: Revolutions per minute
SCA1: Spinocerebellar ataxia type 1
TFR1: Transferrin receptor 1
Th: Thalamus
UCPs: Uncoupling proteins
