# Supplementary material for: Ucp4 Knockdown of Cerebellar Purkinje Cells Induces Bradykinesia
Source: Mol Neurobiol. 2023 Sep 9;61(2):1119–39. doi: 10.1007/s12035-023-03607-1 (PMC10861399; doi:10.1007/s12035-023-03607-1)
Supplement: Supplementary file 1 — (DOCX 4143 kb) [file 12035_2023_3607_MOESM1_ESM.docx]

**Supplementary information**

**Title:**

***Ucp4* knockdown of cerebellar Purkinje cells induces bradykinesia**

**Running Title:**

***Ucp4* knockdown induces bradykinesia**

**Authors:**

Ya-Yun Wang ^1, 6 *^

Hui Liu ^1, 3 #^,

Shu-Jiao Li ^1 #^,

Ban Feng ^1, 4 #^,

Yun-Qiang Huang ^1^,

Shui-Bing Liu ^5 *^,

Yan-Ling Yang ^2 *^

**Author Affiliations:**

*1. National Teaching Demonstration Center, School of Basic Medicine, Air Force Medical University (Fourth Military Medical University), Xi'an, China*

*2. Department of Hepatobiliary Surgery, Xi-Jing Hospital, Air Force Medical University (Fourth Military Medical University), Xi'an, China*

*3. Department of Human Anatomy, Histology and Embryology, Medical School of Yan'an University, Yan'an, China*

*4. State Key Laboratory of Military Stomatology & National Clinical Research Center for Oral Disease & Shaanxi Engineering Research Center for Dental material and Advanced Manufacture, department of Pharmacy, Air Force Medical University (Fourth Military Medical University), Xi'an, China*

*5. Department of Pharmacology, School of Pharmacy, Air Force Medical University (Fourth Military Medical University), Xi'an, China*

*6. State Key Laboratory of Military Stomatology, School of Stomatology, Air Force Medical University (Fourth Military Medical University), Xi'an, China*

**# These authors have contributed equally to this work**

*** These authors are corresponding authors contributed equally to this work**

*** Corresponding authors:**

*** Ya-Yun Wang,**

Ph. D. & M.D.

Professor

Principle Investigator of Specific Lab for Mitochondrial Plasticity Underlying Nervous System Diseases

Director of National Demonstration Center for Experimental Preclinical Medicine Education

Air Force Medical University (Fourth Military Medical University)

Xi'an 710032, China

E-mail: wangyy@fmmu.edu.cn; 462307338@qq.com

Office Tel: 0086-29-84774104

Mobile: 0086-13679168991

ORCID: 0000-0002-0397-0390

*** Yan-Ling Wang,**

Ph. D. & M.D.

Professor

Department of Hepatobiliary Surgery

Xi-Jing Hospital

Air Force Medical University (Fourth Military Medical University)

Xi'an 710032, China

E-mail: yangyanl@fmmu.edu.cn

Office Tel: 0086-29-84712341

Mobile: 0086-13709246656

ORCID: 0000-0001-8246-1756

*** Shui-Bing Liu,**

Ph. D. & M.D.

Professor

Department of Pharmacology

School of Pharmacy

Air Force Medical University (Fourth Military Medical University)

Xi'an 710032, China

E-mail: liushb1974@aliyun.com

Office Tel: 0086-29-84774555

Mobile: 0086-13891886006

Hui Liu: [liuhui06@fmmu.edu.cn;](mailto:liuhui06@fmmu.edu.cn;) 1239592310@qq.com

Shu-Jiao Li: [lishujiao@fmmu.edu.cn;](mailto:lishujiao@fmmu.edu.cn;) ORCID: 0000-0002-5577-4690

Ban Feng: zuiaifuqin@163.com

Yun-Qiang Huang: [huangyunqiang@fmmu.edu.cn](mailto:huangyunqiang@fmmu.edu.cn)

Shui-Bing Liu: [liushb1974@aliyun.com](mailto:liushb1974@aliyun.com)

Yan-Ling Yang: [yangyanl@fmmu.edu.cn;](mailto:yangyanl@fmmu.edu.cn;) ORCID: 0000-0001-8246-1756

Ya-Yun Wang: wangyy@fmmu.edu.cn; 462307338@qq.com;ORCID: 0000-0002-0397-0390


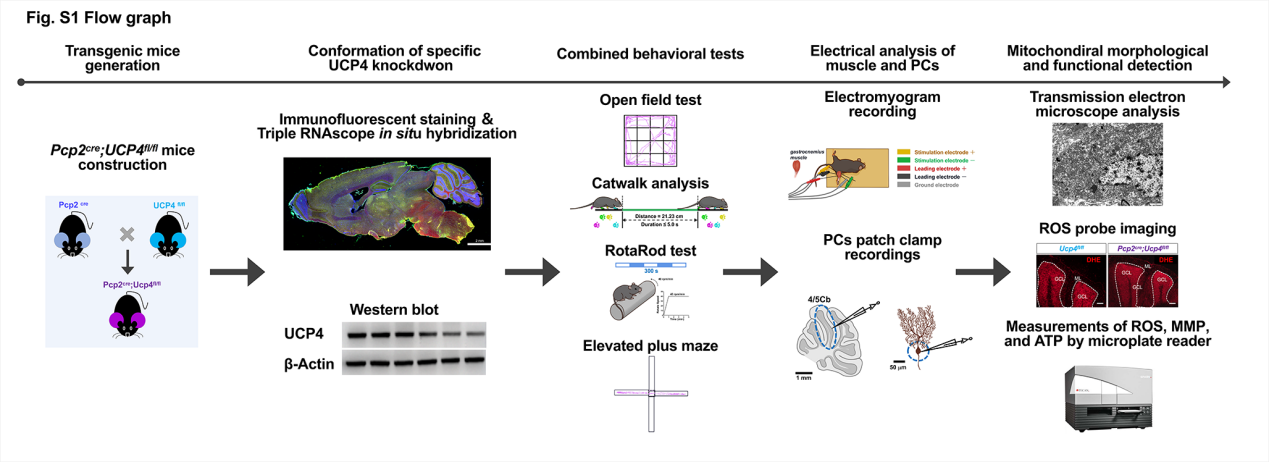


**Fig. S1.** **Study flow chart.**

In this study, Ucp4 knockdown mice were first generated. Then the confirmation of Ucp4 expression knockdown in cerebellar Purkinje cells (PCs) was performed by Western blot, immunofluorescent staining and triple RNAscope in situ hybridization. Further, the combined behavioral tests were used to detect whether *Pcp2^cre^;Ucp4^fl/fl^* mice could display a characteristic bradykinesia in the spontaneous movements. Moreover, the electromyogram recordings detection was used to exclude the possibility of hypotonia in *Pcp2^cre^;Ucp4^fl/fl^* mice. At the same time, the electrical patch clamp recordings was used to detect whether the properties of PCs in *Pcp2^cre^;Ucp4^fl/fl^* mice change. Finally, the state of mitochondrial morphological and mitochondrial function in cerebellum of *Pcp2^cre^;Ucp4^fl/fl^* mice were investigated by transmission electron microscope analysis, reactive oxygen species (ROS) probe imaging, and measurements of ROS generation, mitochondrial membrane potential (MMP), and ATP level by microplate reader.

**
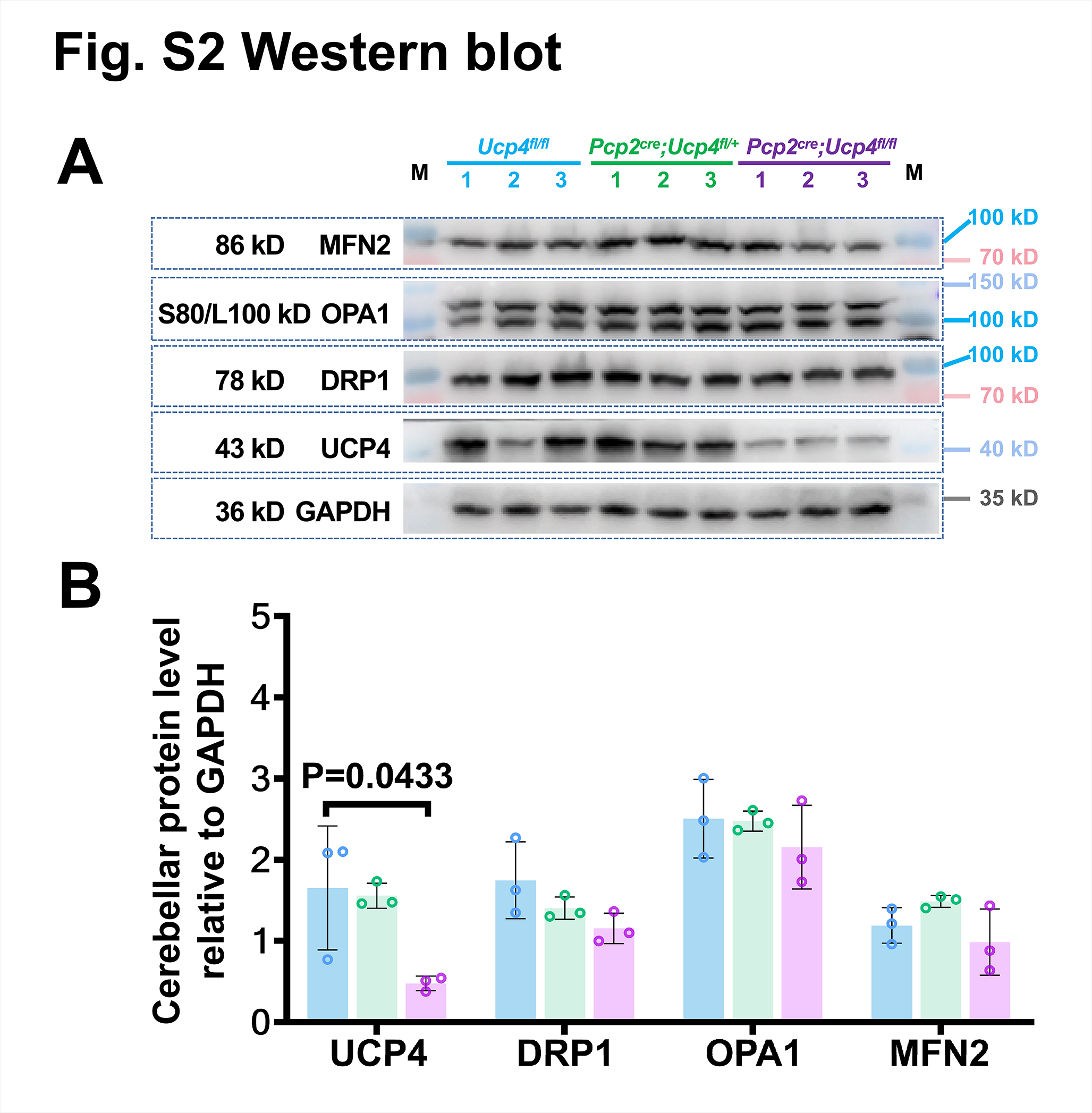
**

**Fig. S2. The protein levels of three other important mitochondrial factors including DRP1, OPA1 and MFN2 had no differences in cerebellum regions among *Ucp4^fl/fl^* mice, *Pcp2^cre^;Ucp4^fl/+^* mice, and *Pcp2^cre^;Ucp4^fl/fl^* mice.**

**A.** Representative Western blot results.

**B.** Quantification for Western blot. The protein expression level of UCP4 relative to the GAPDH internal reference decreased sharply by 70% in *Pcp2^cre^;Ucp4^fl/fl^* mice when compared to the homozygous *Ucp4^fl/fl^* mice. Meanwhile, the expression levels of DRP1, OPA1, and MFN2 relative to GAPDH, showed no significant difference in the three types of transgenic mice.

The data were analyzed by one-way ANOVA and LSD post-hoc test or Kruskal-Wallis test. The data were shown as the mean ± SD; n = 3 mice per group. *P* < 0.05 was considered a statistically significant difference.

DRP1, the mitochondrial fission protein dynamin-related protein 1 located at outer mitochondrial membrane; MFN2, the mitochondrial fusion protein mitofusion 2 located at the outer mitochondrial membrane; OPA1, the mitochondrial fusion protein optic atrophy 1 located at the inner mitochondrial membrane.

**
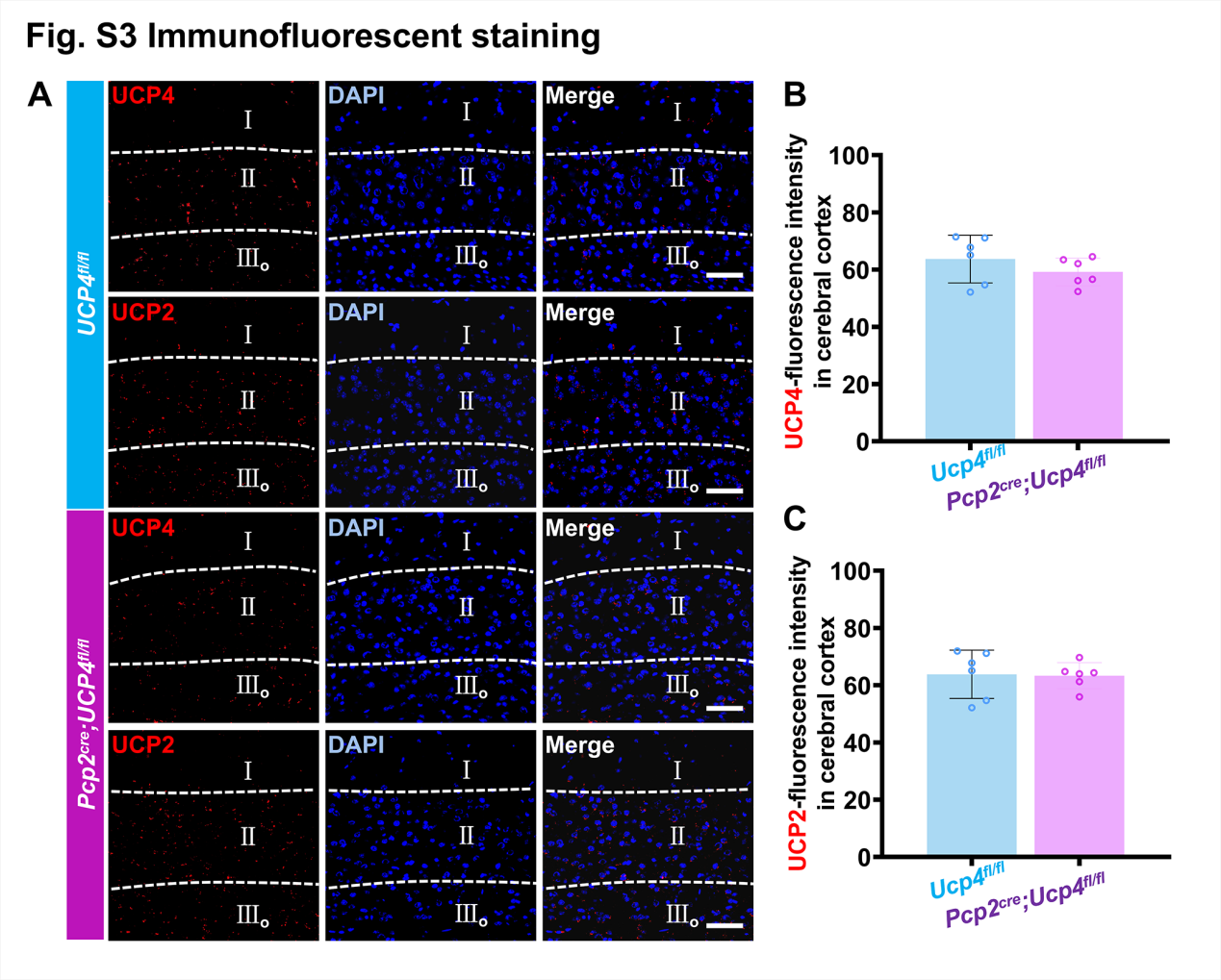
**

**Fig. S3. Immunofluorescent staining confirmed the normal Ucp4 expression in cerebral cortex of *Pcp2^cre^;Ucp4^fl/fl^*** **mice.**

**A.** Confocal images of Ucp4 expression (red) or Ucp2 (red) in three layers including layer I (I), layer II (II) and outer part of layer III (IIIo) in the sagittal sections of cerebellar cortex of *Ucp4^fl/fl^* mice **(top line)** and *Pcp2^cre^;Ucp4^fl/fl^* mice **(bottom line)**. DAPI (blue) is used to locate somatic bodies. Bars = 50 μm.

**B and C.** Quantification of UCP4 **(B)** / UCP2 **(C)** - fluorescence intensity in cerebral cortex.

The data were shown as the mean ± SD. These results were analyzed by unpaired t-test, n = 6 mice per group. *P* < 0.05 was considered a statistically significant difference.

**
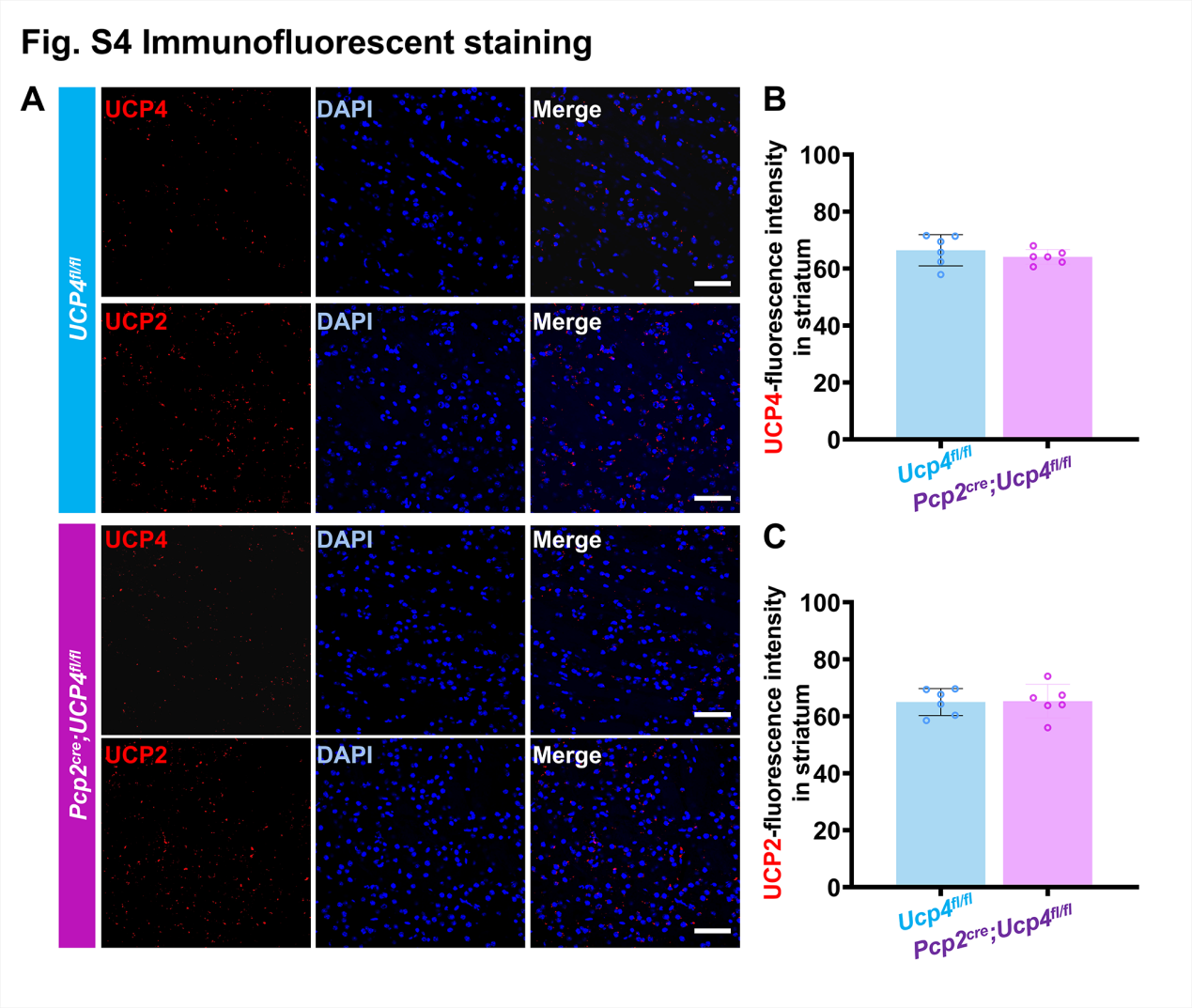
**

**Fig. S4. Double immunofluorescent staining confirmed the normal Ucp4 expression in striatum of *Pcp2^cre^;Ucp4^fl/fl^*** **mice.**

**A.** Confocal images of Ucp4 expression (red) or Ucp2 (red) in the sagittal sections of striatum of *Ucp4^fl/fl^* mice **(top line)** and *Pcp2^cre^;Ucp4^fl/fl^* mice **(bottom line)**. DAPI (blue) is used to locate somatic bodies. Bars = 50 μm.

**B and C.** Quantification of UCP4 **(B)** / UCP2 **(C)** - fluorescence intensity in striatum.

The data were shown as the mean ± SD. These results were analyzed by unpaired t-test, n = 6 mice per group. *P* < 0.05 was considered a statistically significant difference.

**
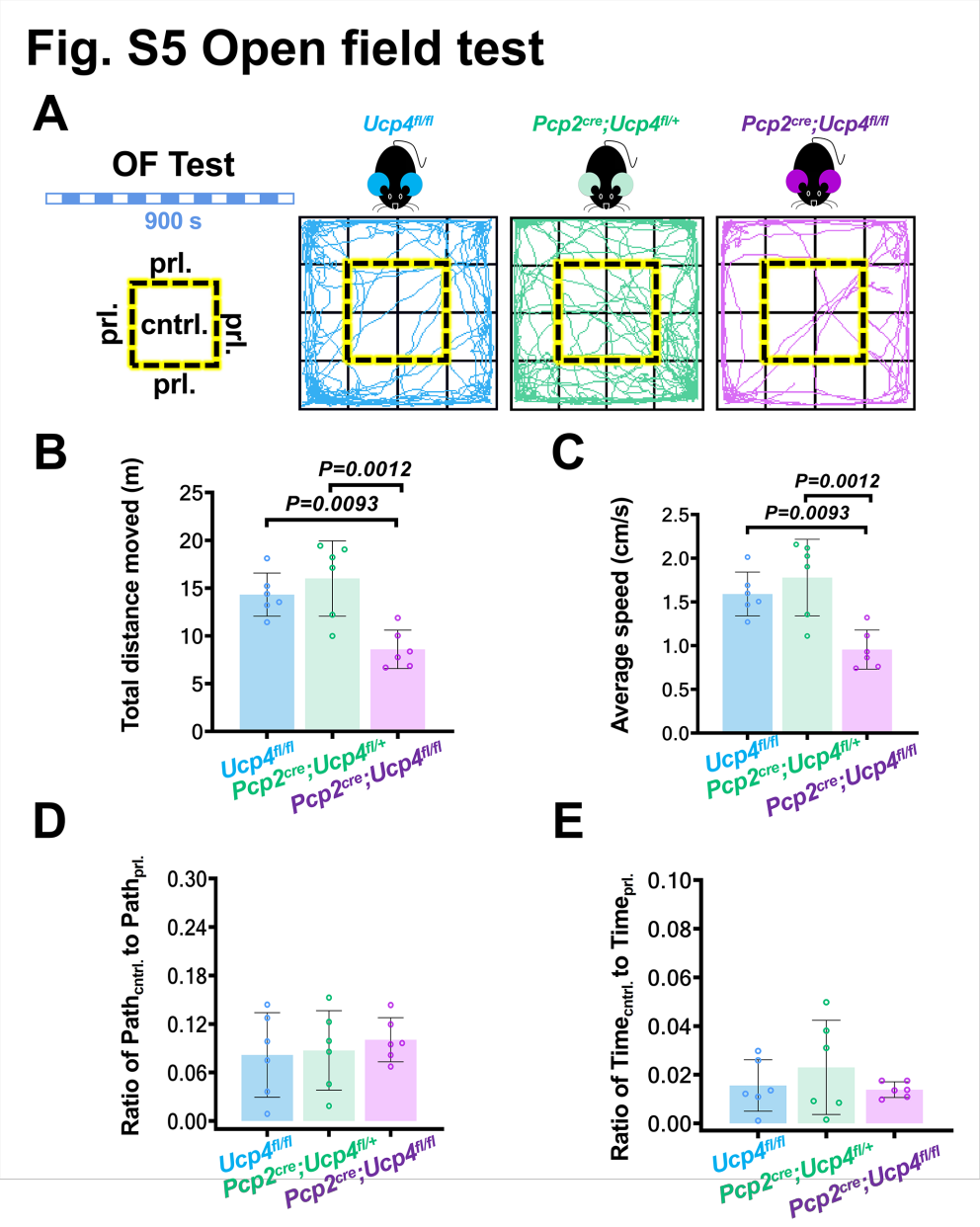
**

**Fig. S5. *Ucp4^fl/fl^* mice and *Pcp2^cre^;Ucp4^fl/+^* mice had no difference in performances by open field (OF) test.**

**A.** Representative traces of the open field (OF) test. The light and dark segmented lines showed the 900-s duration of the OF test. The closed and open arms, as well as the central platform were marked. Four central squares and 12 peripheral squares were marked.

**B–E.** Quantification of the OF test. The total distance **(B**, m**)** and average speed **(C**, cm/m**)** reduce by approximately 40% in *Pcp2^cre^;Ucp4^fl/fl^* mice, compared to *Ucp4^fl/fl^* mice. There was no difference in the Ratio of Path_cent._ to Path_prl._ (**C)** or Ratio of Time_cent._ to Time_prl._ (**D)** among three groups.

Statistical analysis was performed by one-way ANOVA and LSD post-hoc test or Kruskal-Wallis test. The data were shown as the mean ± SD, n = 6 mice per group.
